# Caging Polycations: Effect of Increasing Confinement on the Modes of Interaction of Spermidine^3+^ With DNA Double Helices

**DOI:** 10.3389/fchem.2022.836994

**Published:** 2022-02-25

**Authors:** Tudor Vasiliu, Francesca Mocci, Aatto Laaksonen, Leon De Villiers Engelbrecht, Sergiy Perepelytsya

**Affiliations:** ^1^ Centre of Advanced Research in Bionanoconjugates and Biopolymers “Petru Poni” Institute of Macromolecular Chemistry, Iasi, Romania; ^2^ Dipartimento di Scienze Chimiche e Geologiche, Cagliari University, Cagliari, Italy; ^3^ Division of Energy Science, Energy Engineering, Luleå University of Technology, Luleå, Sweden; ^4^ Division of Physical Chemistry, Department of Materials and Environmental Chemistry, Arrhenius Laboratory, Stockholm University, Stockholm, Sweden; ^5^ State Key Laboratory of Materials-Oriented and Chemical Engineering, Nanjing Tech University, Nanjing, China; ^6^ Bogolyubov Institute for Theoretical Physics of the NAS of Ukraine, Kyiv, Ukraine

**Keywords:** polyamine, condensation, DNA, counterion, molecular dynamics

## Abstract

Polyamines have important roles in the modulation of the cellular function and are ubiquitous in cells. The polyamines putrescine^2+^, spermidine^3+^, and spermine^4+^ represent the most abundant organic counterions of the negatively charged DNA in the cellular nucleus. These polyamines are known to stabilize the DNA structure and, depending on their concentration and additional salt composition, to induce DNA aggregation, which is often referred to as condensation. However, the modes of interactions of these elongated polycations with DNA and how they promote condensation are still not clear. In the present work, atomistic molecular dynamics (MD) computer simulations of two DNA fragments surrounded by spermidine^3+^ (Spd^3+^) cations were performed to study the structuring of Spd^3+^ “caged” between DNA molecules. Microsecond time scale simulations, in which the parallel DNA fragments were constrained at three different separations, but allowed to rotate axially and move naturally, provided information on the conformations and relative orientations of surrounding Spm^3+^ cations as a function of DNA-DNA separation. Novel geometric criteria allowed for the classification of DNA-Spd^3+^ interaction modes, with special attention given to Spd^3+^ conformational changes in the space between the two DNA molecules (caged Spd^3+^). This work shows how changes in the accessible space, or confinement, around DNA affect DNA-Spd^3+^ interactions, information fundamental to understanding the interactions between DNA and its counterions in environments where DNA is compacted, e.g. in the cellular nucleus.

## 1 Introduction

The most common structural organization of the DNA macromolecule in cells consists of two polynucleotide chains wound in a double helix (Watson and Crick, 1953). The nucleotides occurring in DNA are adenine (A), thymine (T), guanine (G) and cytosine (C), the sequence of which codes genetic and structural information. In aqueous solution at physiological pH, each nucleotide is negatively charged due to deprotonation of the phosphate groups. The phosphates, together with the sugar rings, constitute the DNA backbone, which is the region most exposed to the solvent in DNA helices. The double helix is stabilized by positively-charged ions in the solvent media (counterions), typically metal ions (Na^+^, K^+^, Mg^2+^) and charged organic molecules like polyamines (PAs) (Franklin and Gosling, 1953; Ames and Dubin, 1960; Blagoi et al., 1991; Maleev et al., 1993; Mocci and Laaksonen, 2012; Mocci et al., 2021). The counterions bind to different regions of the double helix (minor and major grooves, phosphate groups), and these interactions are essential for the organization of the macromolecule in high order structures (Mocci and Laaksonen, 2012). In the cells of living organisms, DNA is organized in a highly compact form, wrapping around histone proteins in the nucleosome core particles (NCPs), which further assemble, forming chromatin fibers (Saenger, 1984; Schlick, 2002). The PAs are involved in the neutralization of DNA in chromatin and are essential for NCPs formation (van Dam et al., 2002; Korolev et al., 2012). Multivalent counterions are present also in viruses, neutralizing the negatively-charged DNA and RNA macromolecules, and allowing them to pack densely inside the small volume of the viral capsid (Ames and Dubin, 1960; Roos et al., 2007; Carrivain et al., 2012; Mounce et al., 2017; Firpo and Mounce, 2020). The interactions of positively-charged PAs with DNA have a significant biological effect, and are also involved in some emerging biotechnological applications (Korolev et al., 2001; D’Agostino, 2018; Perepelytsya et al., 2019; Mocci et al., 2021; Vasiliu et al., 2021). The study of the role of PAs in the structural organization of DNA in living organisms is of paramount importance for the understanding of key biological functions.

While the binding of monoatomic metal ions to DNA have been extensively studied, and there exists a vast amount of data showing the character of their interaction with nucleic acids (NAs) (Saenger, 1984; Blagoi et al., 1991; Maleev et al., 1993; Young et al., 1997; McConnell and Beveridge, 2000; Mocci and Saba, 2003; Mocci et al., 2004; Ponornarev et al., 2004; Várnai and Zakrzewska, 2004; Marincola et al., 2009; Mocci and Laaksonen, 2012; Lavery et al., 2014; Pasi et al., 2015; Atzori et al., 2016; Dans et al., 2016; Perepelytsya, 2018; Zdorevskyi and Perepelytsya, 2020), the binding of molecular counterions to NAs have been less studied. PAs are known to affect the dynamics and structure of the DNA double helix, inducing condensation (Chattoraj et al., 1978; Gosule and Schellman, 1978; Bloomfield, 1996; Kornyshev et al., 2007; Estévez-Torres and Baigl, 2011). Experimental data show that counterions with charge greater than 2 induce compaction of DNA; this effect depends on the type and concentration of the counterions (Estévez-Torres and Baigl, 2011). The PAs spermidine^3+^ and spermine^4+^ induce the condensation of DNA when the PA concentration is sufficient to completely neutralize the NA. At low concentration of spermine^4+^, or some of its isomers, the effects of enhancement and inhibition of gene activity were established for the case of low and high concentrations the PAs, respectively (Kanemura et al., 2018; Kitagawa et al., 2021). The problem of understanding the molecular mechanisms of PA-induced DNA condensation belongs to the frontiers between chemistry, biology and physics.

To describe the DNA condensation induced by multivalent metal ions and PAs, different theoretical models have been proposed [see the reviews (Bloomfield, 1996; Kornyshev et al., 2007)]. The collapse of the DNA macromolecular chain was considered as a coil-globule transition in a statistical mechanics approaches (Post and Zimm, 1979) that was developed further for different cases of the DNA state [see the review (Bloomfield, 1997)]. The attraction between DNA double helices, eventually resulting in condensation, was shown to arise due to the interaction between the polyanionic macromolecules with the mobile counterions, which in the case of ion charge ≥3 form a structured system between two macromolecules resembling Wigner crystal (Rouzina and Bloomfield, 1996). Such a model describes the character of DNA-DNA attraction in the case of small multivalent ions, but the attraction that appears in the case of some bivalent metal ions (Mn^2+^ or Ca^2+^) and elongated PA molecules are not clear (Kornyshev et al., 2007). The localization of counterions in the grooves of the double helix are taken into consideration in the electrostatic “zipper” model (Kornyshev and Leikin, 1999). In this model, the enhanced attraction between different helices appears due to the juxtaposition of negatively charged phosphate groups of the double helix backbone with the positively charged counterions in the DNA grooves. In spite of great efforts by scientists in developing these theoretical models, the microscopic mechanism of DNA condensation induced by flexible highly charged PAs (spermidine^3+^ and spermine^4+^) and their aggregates is not yet clear. Many aspects of these processes are still to be determined, such as how the interactions are dependent on the concentration of the PA, or by the particular nucleotides sequence, or by the distance between DNA molecules (or portion of the same long DNA fragment). In this context, molecular dynamics (MD) simulation methods can be a powerful tool to obtain detailed information.

The first MD simulations of DNA with PAs showed that these counterions strongly bind to the double helix, substituting monovalent metal ions (Korolev et al., 2001, 2004; van Dam et al., 2002). As shown also by other simulations, the modes of interaction between PAs and DNA double helix are governed by noncovalent interactions and are extremely variable, affected by the PAs charge and length (Korolev et al., 2001, 2004; van Dam et al., 2002; Bignon et al., 2017; Perepelytsya et al., 2019), and yet to be properly classified. Interacting with DNA, the PAs induce changes in the double helix structure, in particular narrowing of the minor groove (Korolev et al., 2004). While the interactions of PAs with the DNA were for a long time considered non-sequence specific, recent experiments (Patel and Anchordoquy, 2006; Kabir and Suresh Kumar, 2013) and MD simulations (Perepelytsya et al., 2019; Mocci et al., 2021) have shown that putrescine^2+^, spermidine^3+^, and spermine^4+^ prefer to be localized in the DNA minor groove of the AT-rich regions. The preferential localization of PAs in the minor groove of the double helix is modulated by the sequence of nucleotides determining the natural narrowing of the minor groove. The aggregation of DNA induced by PAs was found to be stabilized by PA bridges formed in different regions between the double helices (Dai et al., 2008). MD simulation studies of DNA arrays with spermine^4+^ also revealed the sequence-specific effects of PA interactions with DNA and the formation of PA cross-links between two double helices (Yoo and Aksimentiev, 2016; Yoo et al., 2016). The spermidine^3+^ cross-linking between two DNA 22-mers in water solution was observed in a recent MD simulation (Mocci et al., 2021).

The goal of the present work is to study how the spatial organization and dynamics of spermidine^3+^ (Spd^3+^) molecules at the interface between two DNA fragments depends on the separation between the DNA helices. To tackle this problem, atomistic MD simulations were performed for three model systems, each consisting of two 22-base-pairs-long DNA double helices restrained at different interhelix separation distances. A nucleosomal 146-base-pairs-long DNA fragment, with coordinates taken from an X-ray structure of an NCP, was also simulated, constraining it to the experimental geometry and helix separation. The DNA in the NCP system constitutes an ideal model of DNA compaction at a level found in cellular environments. It is important to note that in the NCP both the separation between the DNA helices and the high density of the positive charge of the histones are expected to influence the Spd^3+^/DNA interactions. To disentangle the effects of the DNA separation from those due to the presence of the positively charged core of the NCP, and to allow a comparison with the other parallel double helix models used in this investigation, we chose to omit the protein core. Its effect will be addressed in future studies. The details of the MD simulations are described in Section 2. The structural organization, and the dynamics, of Spd^3+^ around the DNA double helices are analyzed and discussed in Section 3.

The MD simulations show strong attraction between the DNA helices induced by Spd^3+^ and highlight how the increasing confinement of the PA molecules, obtained by decreasing the distance between DNA helices, affect their structural and dynamical behavior.

## 2 Methods

**FIGURE 1:**
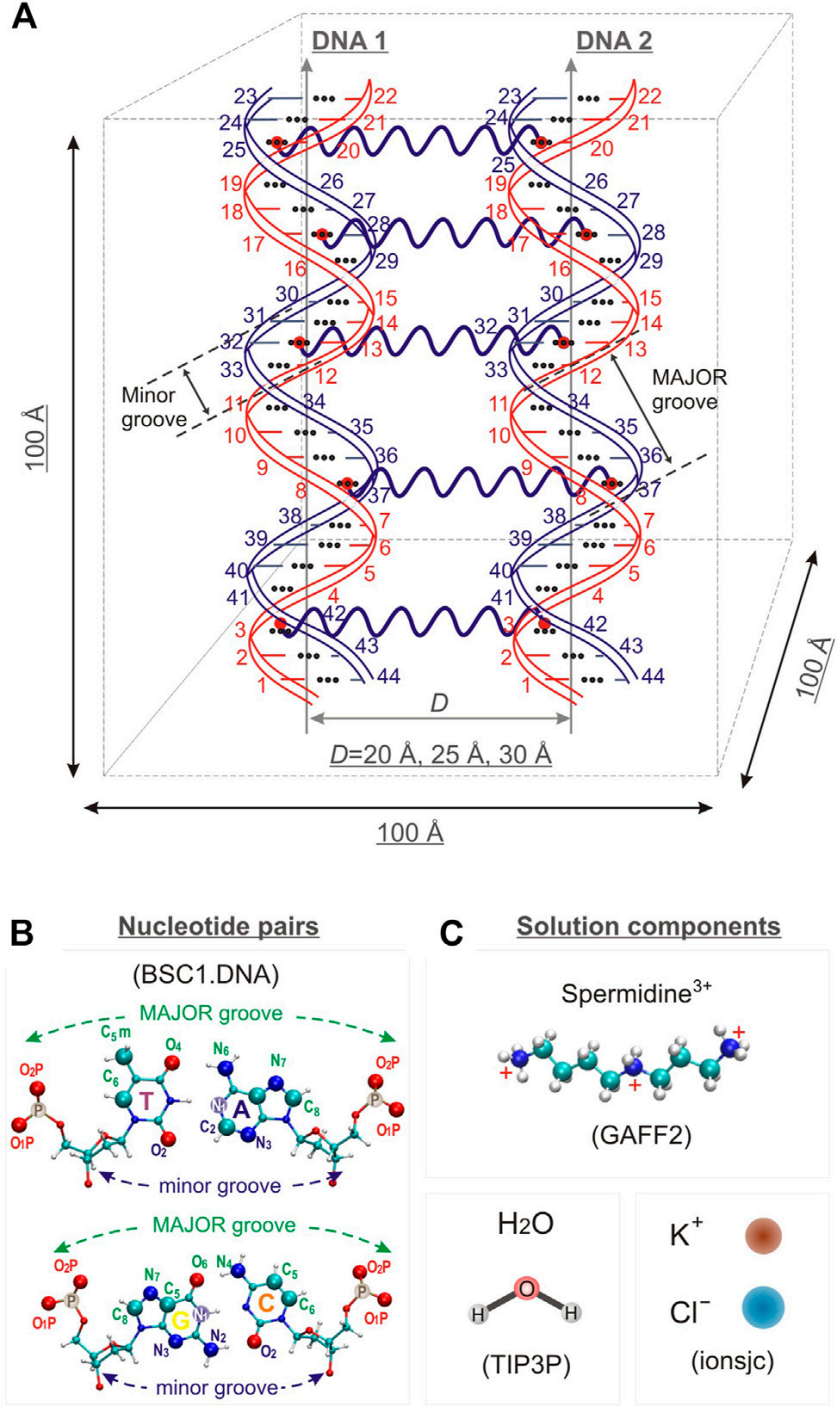
**(A)** Schematic representation of the simulation box for systems DD-20 Å, DD-25 Å, DD-30 Å, together with the numbering of the nucleotide bases, and the indication of the positioning of the restraints used in the simulation to maintain the initial relative orientation and distance between the DNA helices. **(B)** T-A and C-G base pairs with the label of the atoms used as reference in the analysis. **(C)**. Schematic representation of the structure of the solvent and counter- and co-ions of DNA. The force fields used to describe the components are also indicated.

**FIGURE 2:**
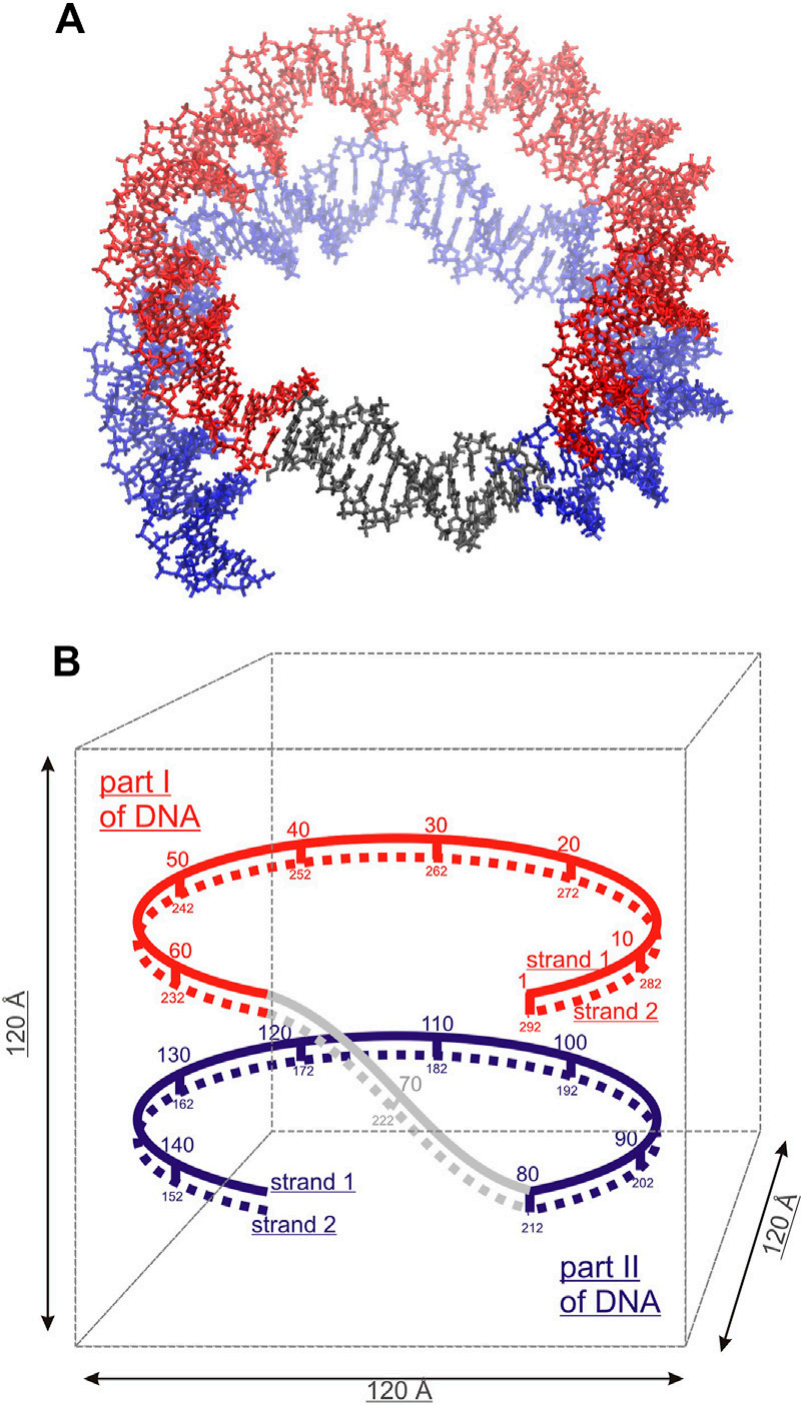
Simulated system of nucleosomal DNA. **(A)** Licorice representation of the nucleosomal DNA: red and blue colors indicate the overlapping parts of the DNA double helix, non-overlapping DNA colored grey. **(B)** Schematic structure of the nucleosomal DNA in the simulation box. The numbering of the nucleotide pairs is indicated.

Three model systems, each containing two identical DNA molecules consisting of 22 base pairs (bps) were constructed by placing the DNA molecules parallel to each other at distances between their axis of 20, 25 and 30 Å, respectively; Spd^3+^ and water molecules were then added. The systems will be referred to as DD-20Å, DD-25Å, and DD-30Å. In addition, to compare the results from the model systems described above with an experimental parallel DNA helix, we performed a simulation of a nucleosomal DNA sequence, 146 bps long, with configuration taken from an experimental NCP X-ray structure, where it is known that the wrapping of DNA around the histones leads the two segments of the duplex to align in parallel (for approximately 66 bps). Schematic representations of the simulation boxes with two DNA duplexes and the system with nucleosomal DNA are shown in Figures 1, 2, respectively. The composition of each system is detailed in Table 1.

**TABLE 1:** Simulation box details.

System name	Number of DNA molecules	Number of bps	Distance between DNA segments^a^	Number of Spd^3+^ molecules	Number of water molecules	Number of ions (K^+^/Cl^−^)
DD-20Å	2	22	20 Å	30	31,308	84/90
DD-25Å	2	22	25 Å	30	31,308	84/90
DD-30Å	2	22	30 Å	30	31,308	84/90
Nucleosome	1	146	25–28 Å	100	50,583	156/166

a In the case of nucleosomal DNA, the reported distance range refers to the separation between the part I and part II, as defined in Figure 2.

The initial structure of Spd^3+^ was built with the Avogadro software (Hanwell et al., 2012; Avogadro, 2018), and the corresponding general AMBER force field (GAFF2) parameters (Wang et al., 2004) were generated with the Antechamber software comprised in the AmberTools 18 package (Case et al., 2018). The atomic partial charges of the Spd^3+^ molecule were calculated using the RESP methodology and are presented in Supplementary Figure S1.

The nucleic acid builder (NAB) tool contained in the AmberTools 18 package was used to create the 22 bps DNA in the Arnott *B*-DNA canonical structure with the sequence d(CGC​GAA​TTC​GCG​CGA​ATT​CGC​G), containing two motifs of the Drew-Dickerson dodecamer (Drew et al., 1981) sequence, with two A-tracts underlined. This sequence, containing both A-tracts and CG-rich regions, constitute an important model system for the study of interaction of PAs with the DNA double helix (Perepelytsya et al., 2019; Mocci et al., 2021). The structure of DNA in an NCP was retrieved from the experimental structure of Tsunaka et al. (Tsunaka et al., 2005) as deposited in the Research Collaboratory for Structural Bioinformatics Protein Data Bank (ID: 2cv5). For a comparison with the other simulated systems, the protein core of the NCP was omitted in the simulations, and the DNA structure was constrained to the experimental structure throughout the entire simulation.

The BSC1. DNA (Ivani et al., 2016) AMBER force field was used for DNA, the TIP3P model was used for water (Jorgensen et al., 1983), while the ionsjc parameters optimized for this water model were used for K^+^ and Cl^−^ ions (Joung and Cheatham, 2008).

All simulations were performed using the GROMACS 2020 software package (Abraham et al., 2015). The simulations were done at constant temperature and pressure. The temperature was set to 298 K, and was controlled using the Nosé-Hoover thermostat (Nosé, 1984; Hoover, 1985). The pressure was set to 1 bar and was controlled using the Parrinello-Rahman barostat (Parrinello and Rahman, 1981). The length of all bonds between hydrogen and other atoms was constrained using the LINCS algorithm (Hess et al., 1997). The smooth particle mesh Ewald method (Darden et al., 1993) was used to calculate the long-range electrostatic interactions. The cut-offs for the switching and the long-range interactions were set to 10 Å, and the Fourier spacing was set to 1.2 Å. The length of each production simulation trajectory was 500 ns.

To restrain the distance between DNA molecules in the DD-20Å, DD-25Å and DD-30Å systems, we used the center-of-mass (COM) pulling method with an umbrella potential (Abraham et al., 2015). In detail, we placed five harmonic springs between the N_1_ atoms of parallel bps in the two DNA molecules, as depicted in Figure 1A. The main advantage of using this restraining method, instead of the classic position restraints, is that the DNA molecules can now move freely (move around the simulation box, rotate around their own helix axes, compress, undergo sequence dependent structural modifications, elongate or bend). We chose this restraining method to eliminate any artifacts in the mode of interaction with the Spd^3+^ that could arise due to the rigid nature of the position restraints. These restraints allowed us to keep the DNA fragments parallel to each other and inhibit the rotation between their axis. We kept the fragments parallel for two reasons: firstly, the parallel conformation occurs naturally in nucleosomes and DNA fibers; secondly the parallel conformation provides a greater area with a controlled distance between the DNA fragments, which translates into a higher number of caged Spd^3+^, which in turn increases the sampling of the conformational space, reducing the need to repeat or greatly extend the simulations. For the nucleosomal DNA simulation we used the classic position restraints, restraining all heavy atoms, because it was important to keep the DNA fragment in the specific experimental “nucleosome conformation”. Due to its size and specific conformation, using COM pulling restraints on this DNA fragment would have implied using at least 15 springs, and the fine tuning of the parameters describing all these springs, to have them maintain the nucleosome conformation, proved to be an unfeasible and inefficient task. The snapshots of the nucleosomal DNA are shown in Supplementary Figure S5.

The analysis of Spd^3+^ distribution was done in terms of radial distribution functions (RDFs) calculated for the PA heavy atoms and selected atoms of the DNA double helix:

$$g\left(r\right)=\underset{\Delta r\to 0}{\lim}\frac{p\left(r\right)}{4\pi r^{2}\Delta rN_{p}/V}$$ (1)

where *p(r)* is the average number of particles that is found at the distance *r* within a shell with thickness *Δr*; *N*_*p*_ is the number of pairs of selected atoms for which the RDF is calculated, and *V* is the system volume. In our calculations, the shell thickness *Δr* has been taken equal to 0.1 Å. The average number of particles within a given distance *r* (coordination number) can be determined by the direct integration of the RDF.

RDFs for Spd^3+^ in the minor and major grooves (RDF_MIN_ and RDF_MAJ_) were calculated between all heavy atoms of Spd^3+^ and the atoms N_3_, N_2_, O_2_, and C_8_, N_7_, C_5_, O_6_, N_4_, C_5_, C_6_ of the nucleotide bases, for the minor and major grooves respectively. The distribution of Spd^3+^ with respect to the phosphate groups was characterized by the RDF_PH_ computed for the Spd^3+^ heavy atoms, with the oxygen atoms O_1_P and O_2_P of the phosphate groups taken as reference atoms (Figure 1B). To analyze the orientation of the DNA double helices with respect to each other, the RDFs (hereafter RDF_DD_s) of the P atoms of DNA1 with respect to the P atoms of DNA2, and vice versa, were calculated. In the case of nucleosomal DNA, where the parallel segments of DNA belong to the same molecule, we indicate with DNA1 and DNA2 the parallel segments with the nucleotide pairs 1–67 and 80–146, respectively (Figure 2B).

The visual inspection of the simulated systems and the analysis of the simulation trajectories were performed using the VMD program and plug-ins implemented in the software package (William et al., 1996). In particular, to characterize the structure of the DNA double helix, the parameters of the minor and major grooves were calculated using VMD plug-in do_x2dna (Lu and Olson, 2003; Kumar and Grubmüller, 2015). The groove widths were calculated according to the definition of El Hassan and Calladine (El Hassan and Calladine, 1998). The RDFs were calculated using the VMD plug-in (Levine et al., 2011) implemented in VMD. The quantitative analysis of the distribution and residence time of Spd^3+^ was performed with VMD using in-house scripts.

## 3 Results

### 3.1 Interaction Between the DNA Helices

**FIGURE 3:**
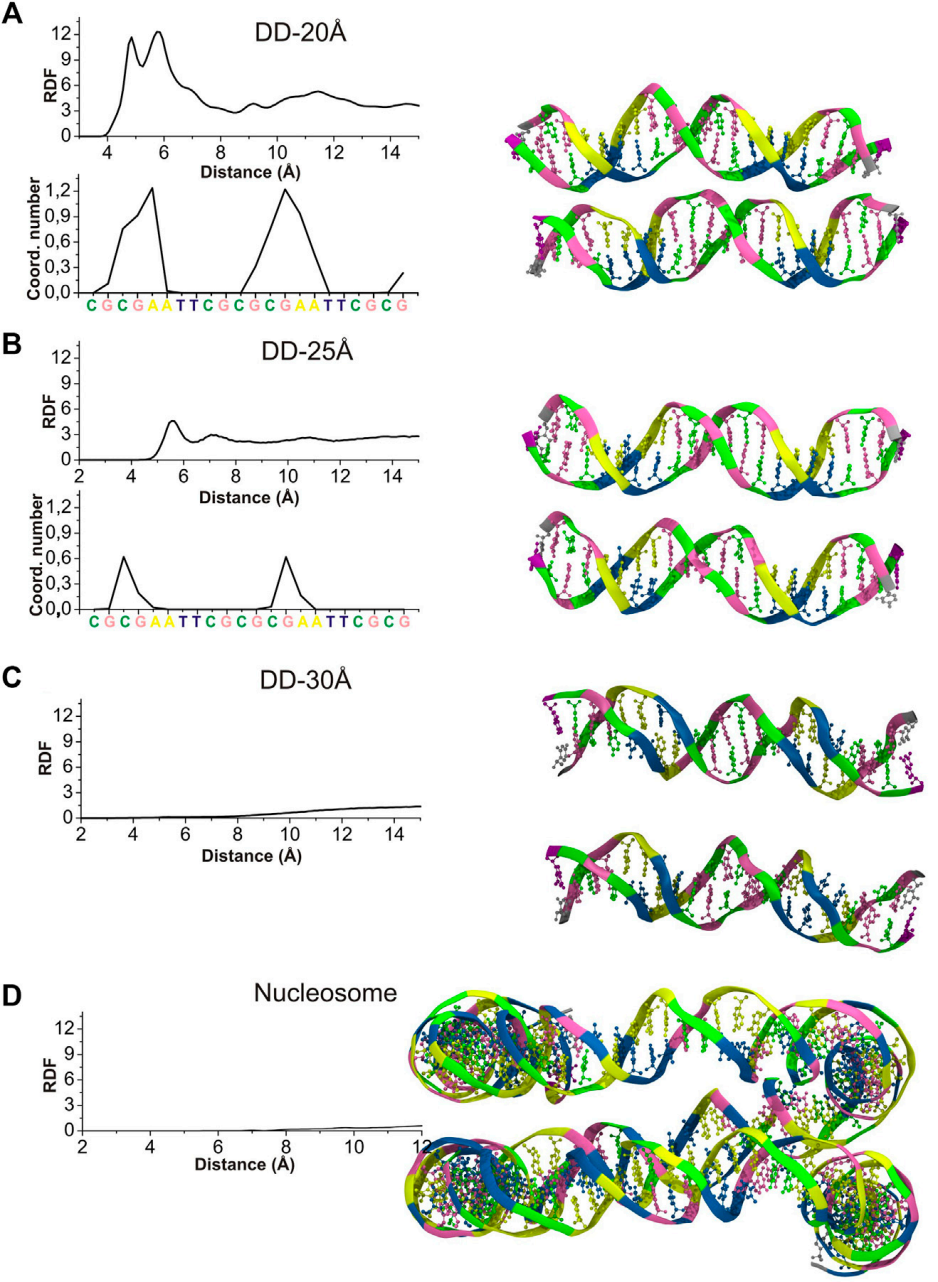
Left: RDF_DD_s (continuous lines) calculated between the P atoms of phosphate groups belonging to different DNA molecules (or parallel segments for nucleosome DNA), and the values of integrals of the RDFs calculated for each individual phosphate group up to 6.4 Å, revealing the sites were the DNA-DNA contacts are most probable. **(A)** System DD-20 Å **(B)** System DD-25 Å. **(C)** System DD-30 Å **(D)** Nucleosome DNA system. Right: The snapshots from the MD simulation of each system are represented at the right side of the corresponding RDF. Coloring scheme: A, yellow; T, blue; C, green; G, pink. Spd^3+^, water and ions have been omitted for clarity.

To characterize the mutual orientation of the DNA helices, the RDFs between the P atoms of one DNA molecule with respect to the P atoms of the other DNA molecule, were calculated for all simulated systems. These RDFs will be indicated as RDF_DD_s in the text and are shown in Figure 3. To identify the nucleotides among which intermolecular contacts are most probable, the RDF calculated for each P atom was integrated up to 6.4 Å, to obtain the coordination number (CN) (Figure 3).

The RDF_DD_s represented in Figure 3 are obtained averaging the RDF_DD_s for the two parallel helices (or DNA portions in case of nucleosomal DNA). The RDF_DD_s calculated separately for each DNA molecule are shown in Supplementary Figure S6. The first maximum of RDF_DD_s of the systems DD-20Å and DD-25Å corresponds to close contacts between the parallel double helixes. This type of interaction, detailed in the following, involve the NH_2_^+^ or NH_3_^+^ group(s) of one or more Spd^3+^ molecules bridging the O atoms of the phosphate groups of the two double helices (O-HNH-O contacts). In the case of the system DD-20Å, where the distance between the two helices is the smallest among the simulated systems, the RDFs are characterized by a maximum splitting into two sharp peaks at about 5 Å and 6 Å, and a minimum at about 8 Å (Figure 3A). The broad band from 8 Å to 14 Å is due to other phosphate groups of the DNA chains; this band is not informative for the analysis of DNA-DNA interaction and is not considered further. The RDF_DD_s for the system DD-25Å have a rather regular form with a prominent maximum at about 5.5 Å and a minimum at ca. 6.5 Å (Figure 3B). As in the case of DD-20Å system, the first maximum arises from the amino group(s)-mediated DNA-DNA contacts, of the O-HNH-O type. This implies that even in the case of systems where the DNA molecules have restraints imposing 25 Å separation between the center of selected base pairs, the presence of Spd^3+^ in the region between the two DNA molecules induces an attraction between them, which, in turn, induces helix bending to achieve the amino group-mediated close contact. The intensity of the first peak is, however, greatly reduced compared to the DD-20Å system. In the case of the system DD-30Å, there are no prominent maxima at short distances, since the macromolecules are held at too large a separation for O-HNH-O DNA-DNA contacts to be formed (Figure 3C). Similar to what was observed for the DD-30Å system, the RDF_DD_s of the nucleosome DNA (Figure 3D), are characterized by very low values at short distances, and a gradual increase with increasing distance, without any relevant maximum. It is important to note that the atoms of nucleosome DNA fragment are fixed in our simulation and its RDF_DD_ describe the distribution that corresponds to the X-ray experimental structure.

To determine whether inter-helix contacts can occur with the same probability at any position of the helices, or if on the contrary some regions are favored, the RDF_DD_s were calculated separately for each nucleotide, and the CNs were calculated using an integration limit of 6.5 Å. The obtained CNs are shown in the insets of Figures 3A,B for DD-20Å and DD-25Å, while for DD-30Å and the nucleosome DNA fragment the helices are too far apart, and no contacts are observed. In the case of the DD-20Å system, the nucleotide-specific CN varies greatly along the double helix, with the highest CNs observed in the regions preceding the A-tracts, including the initial portion of the A-tracts, whereas in other regions there are essentially no contacts between the DNA molecules. The CN peaks are localized in the regions where the minor grooves of DNA1 and DNA2 are facing each other, as shown in the snapshot in Figure 3A, forming a “sandwich structure” of minor groove—Spd^3+^ molecules—minor groove, that will be described in the following sections devoted to the study of the interactions with between DNAs and Spd^3+^. In the DD-25Å system, due to the increased separation, the DNA molecules do not adopt the minor groove-to-minor groove orientation, which leads us to believe that the “sandwich structure” requires a certain separation distance. The contacts between the two helices occur approximately in the same regions as for DD-20Å, with much smaller CNs. In system DD-30Å there are no close contacts between the two DNA molecules, although it must be mentioned that, while fine-tuning the rigidity of the springs to maintain the separation, we noticed that when the two DNA came into contact after more than 500 ns of simulations (due to springs being too lax), we obtained the “sandwich structure”. However, in the present paper we have focused our attention on the portion of the MD trajectories in which the separation between the helices is kept to 20, 25 or 30 Å, since we are interested in how this separation influences the interactions with Spd^3+^. Although the nucleosome system has a typical separation between parallel DNA portions that is intermediate between DD-25Å and DD-30Å, the presence of the minor groove to minor groove orientation is still evident. It must be noted that, while in the DD systems this reciprocal orientation of the helices only appeared after the Spd^3+^ molecules had pulled the two DNA helices closer together, the same orientation is observed in the simulated nucleosomal DNA system, the structure of which was constrained to maintain the crystallographic structure, which did not contain Spd^3+^. Most likely, the positively charged amino groups in the lateral chains of histone protein amino acids are responsible for the occurrence of this orientation in the crystallographic structures of this and other NCPs.

### 3.2 DNA Groove Width

**FIGURE 4:**
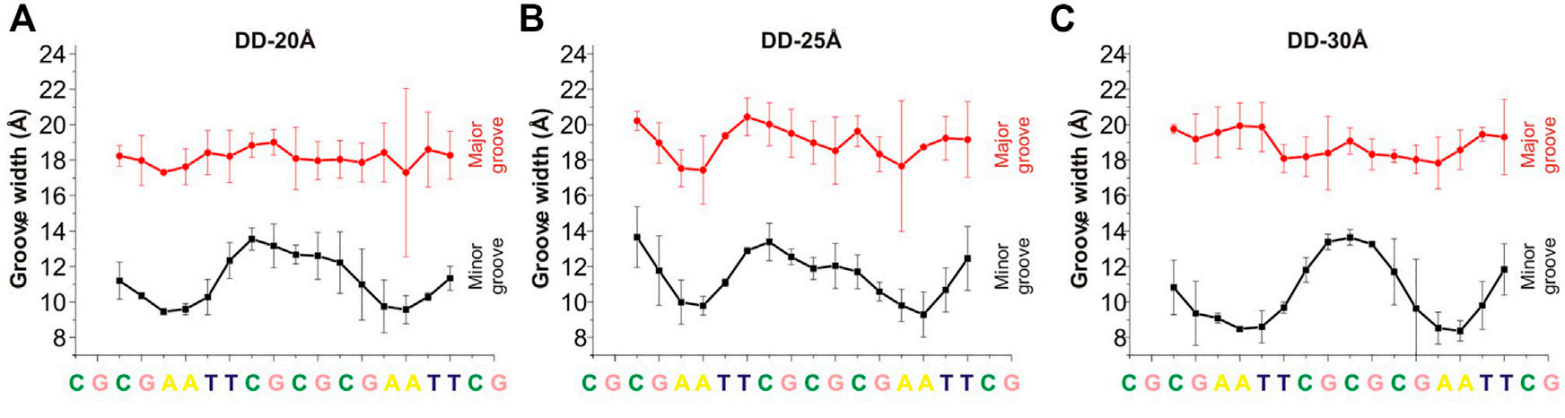
Width of the minor and major grooves for each base pair, in **(A)** DD-20 Å, **(B)** DD-25 Å, **(C)** DD-30 Å. The values of the groove width are averaged over two DNA duplexes. The widths of the grooves for DNA1 and DNA2 are shown in Supplementary Figure S7.

**FIGURE 5:**
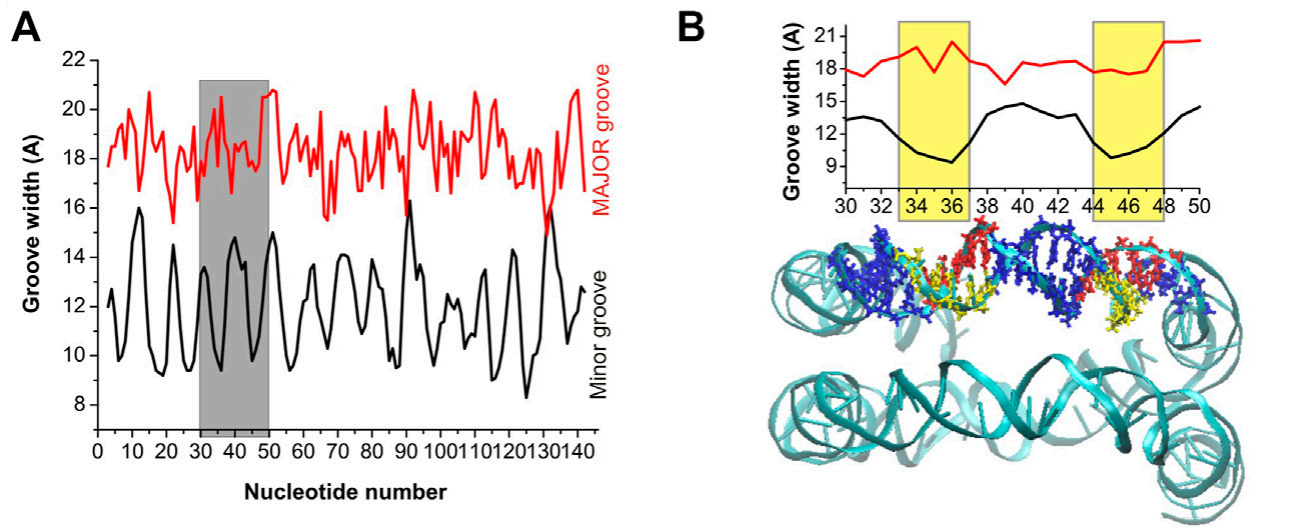
**(A)** Minor and major groove width across the nucleosomal DNA. **(B)** Expansion of the grey band portion of Panel **(A)**, and a representation of the nucleosomal DNA in which the base pairs included in the width calculation are highlighted with a stick representation. The yellow bands, in the groove width plot, mark the region of DNA with the narrowest minor groove, and the corresponding base pairs are highlighted in yellow and red in the DNA snapshot.

An important structural parameter of the DNA double helix structure is the width of the groove, which is dependent on the base pairs sequence and is highly relevant and interconnected with the interactions with the surrounding molecules: a smaller groove width is connected to stronger interactions with PAs (Perepelytsya et al., 2019). In Figures 4, 5 are reported the average values of minor and major groove widths along the double helices, and it can be seen that the minor groove width varies within the range 8–15 Å, while the major groove within 15–22 Å. The fluctuations of the groove width are quantified with the error bars, with exception of the nucleosome DNA system, where a DNA static structure was simulated, and therefore no fluctuation is possible.

In the case of the DD-20Å, DD-25Å and DD-30Å systems, the minor groove width is narrower in the A-tracts regions. Such dependence of the minor groove width on nucleotide sequence is well known and has also been observed previously for systems with PAs (Perepelytsya et al., 2019; Mocci et al., 2021). The dependence of the major groove width on the nucleotide sequence is not as well defined as that of the minor groove.

In the case of the nucleosomal DNA, the minor groove width displays a regular variation along the double helix (Figure 5A). The local minima appear with a periodicity of about 10 bps, and visual inspection of a selected portion of the structure and of the corresponding minor groove width (see Figure 5B) reveals that the minor groove narrowing is observed in the regions corresponding to a close distance between the parallel portions of the DNA. Interestingly, in these regions the minor grooves of the parallel DNA segments face each other, similar to what was observed for system DD-20Å in the region corresponding to the shortest DNA-DNA distances. It should be noted that since in our simulation the structure of the nucleosomal DNA was constrained to its initial geometry, the width of the groove at each base pair cannot vary during the simulation; however, some fluctuation in the groove widths should necessarily occur in the unrestrained NCP DNA, although probably smaller than in the case of the DD-20Å, DD-25Å and DD-30Å systems (Figure 4), due to the constraints imposed in the NCP by the proteins in the core. Therefore, we do not expect great variation in the periodicity of the minor groove width in the real NCP DNA compared to what reported in Figure 5. The variation of the major groove width along the helix is not as regular as for the minor groove, however some correlation between the minima of the major groove and the maxima of the minor groove widths is present (Figure 5A).

### 3.3 DNA-Spd^3+^ Interactions

#### 3.3.1 Distribution and Dynamics in the Interfacial Region Between DNA Molecules

**FIGURE 6:**
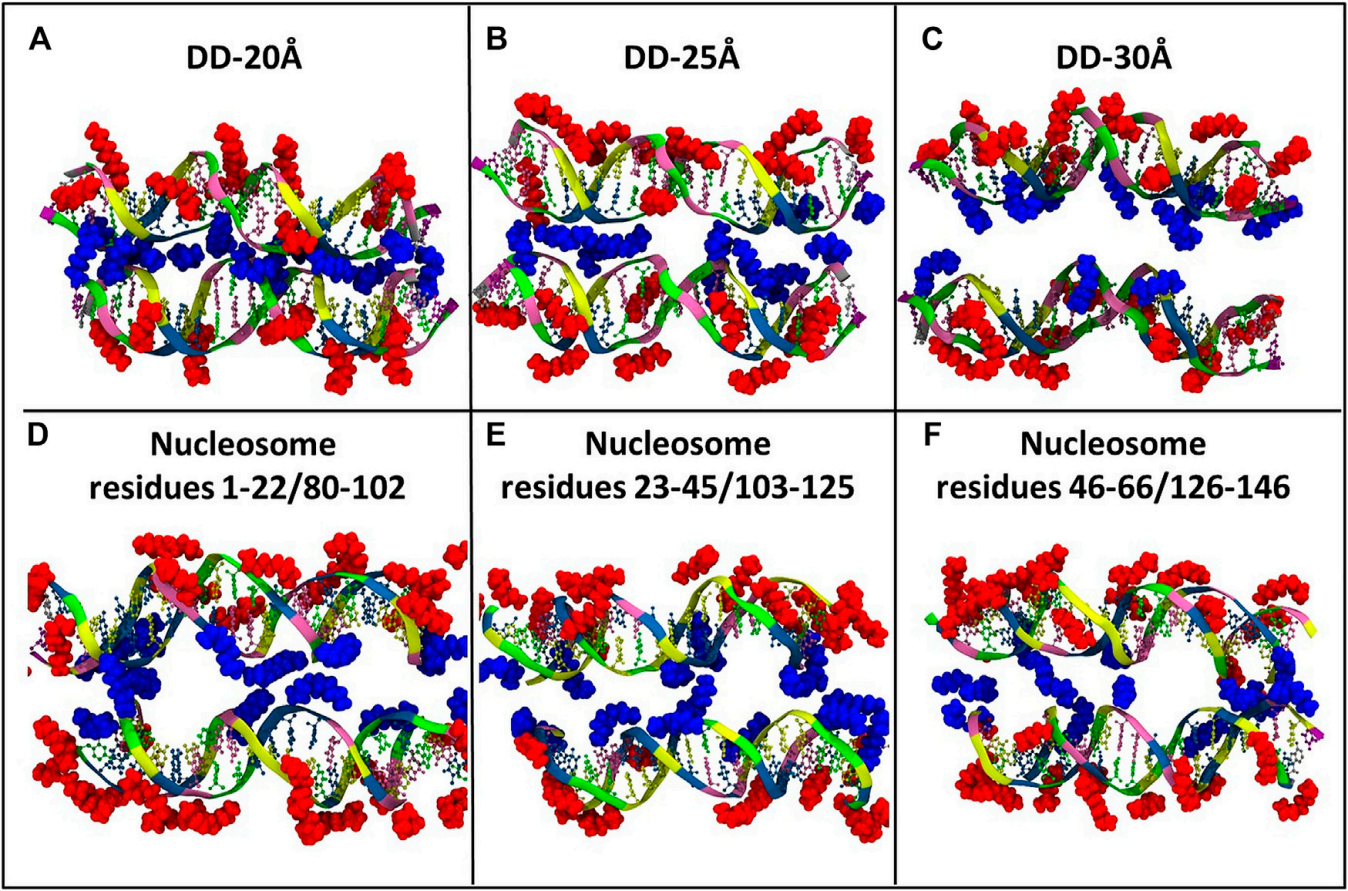
Representative snapshots of the distribution of Spd^3+^ around the two DNA molecules of each system **(A)** DD-20Å, **(B)** DD-25Å, **(C)** DD-30Å, and around the nucleosomal DNA **(D–F)**. To facilitate the visual comparison, the section of the nucleosome system that contains the DNA1 and DNA2 segments was split in three parts with lengths similar to the DD systems. Water and ions are omitted for clarity. Spd^3+^ molecules are colored blue if located in the space between the two DNA molecules (caged), and red if located elsewhere (uncaged).

Visual inspection of the simulation trajectories of the systems DD-20Å, DD-25Å and DD-30Å reveals that all the Spd^3+^ molecules interact with the DNA surface, both in the space between the DNA molecules and in the outer regions. In order to determine the influence of the distance between the DNA molecules on the interaction between DNA and Spd^3+^, we analyzed the simulation snapshots classifying Spd^3+^ molecules as “caged” or “uncaged” as depicted in Figure 6. We considered a Spd^3+^ molecule to be caged if the distance from the central N atom of Spd^3+^ to the center of at least one base pair (i.e. the N_1_ atom in Figure 1) of each DNA molecule is smaller than 15 Å for the DD-20 Å system, 19 Å for the DD-25 Å system, 23 Å for the 30 Å system and 22 Å for the nucleosome system. As it will be shown in the following, the classification of “caged” in our analysis does not necessarily imply that the molecules cannot exit from the region at the interface between the two DNA molecules.

**FIGURE 7:**
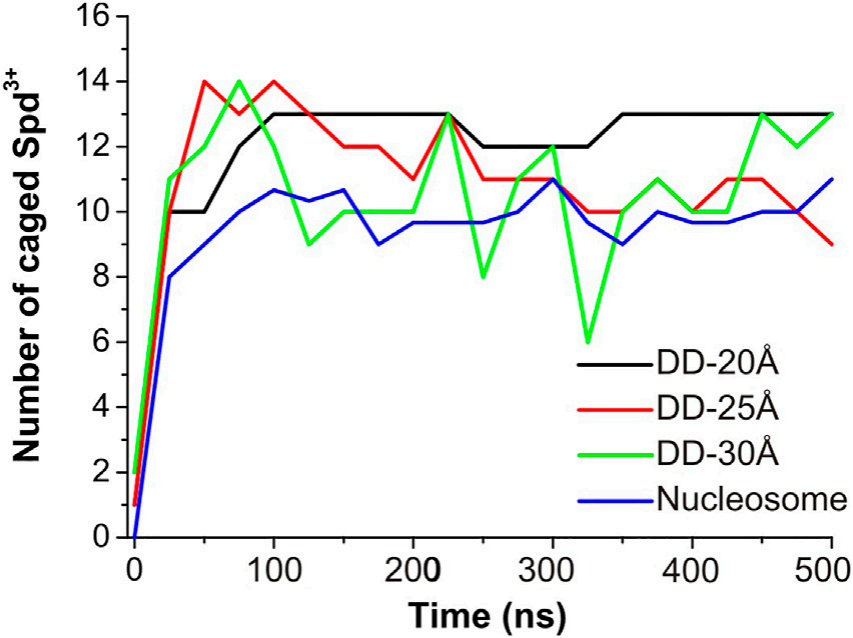
Evolution of the number of Spd^3+^ caged between parallel DNA molecules (or molecular portions). In the case of the nucleosomal system, the total number of Spd^3+^ has been normalized to match the other systems.

To evaluate the effect of DNA-DNA separation on Spd^3+^ - DNA interactions, we calculated at each saved point of the trajectories the number of caged and uncaged Spd^3+^; see Figure 7. It can be seen that in the DD-20Å system, the number of caged molecules reaches a plateau value of 12–13 after t = 100 ns. In the DD-25Å system we notice a maximum of 14 caged Spd^3+^ in the first 100 ns, followed by a fast decrease that results in a fluctuation between 9–11 molecules in the second part of the simulation (250–500 ns). Notably, the fluctuations are much greater than in DD-20A system, where the number of caged molecules varies by a maximum of one unit after the plateau was reached. It should also be considered that the region between the two DNA fragments increases its volume with increasing distance between the DNA helices, and thus the local concentration of Spd^3+^ molecules between the helices decreases significantly. In the DD-30Å system where the distance between the two DNA molecules is further increased, the number of caged Spd^3+^ fluctuates even more over the duration of the simulation, between a maximum of 14 at t = 75 ns and a minimum of 6 at t = 325 ns. Considering that the volume of the region between the two DNA molecules is larger compared to that in the system DD-25Å, the density of Spd^3+^ continues to decrease, and the instantaneous number of caged molecules is much more variable than in the other cases. The variation in the number of caged Spd^3+^ in the nucleosome DNA system closely resembles that in the DD-25Å system; since the DNA size in the nucleosome system is larger, the number of Spd^3+^ has been normalized to allow comparison with DD-25Å, by dividing the instantaneous number of Spd^3+^ by 3 (since the DNA length of each of the nucleosome DNA parallel portions is thrice that of the other systems).

**FIGURE 8:**
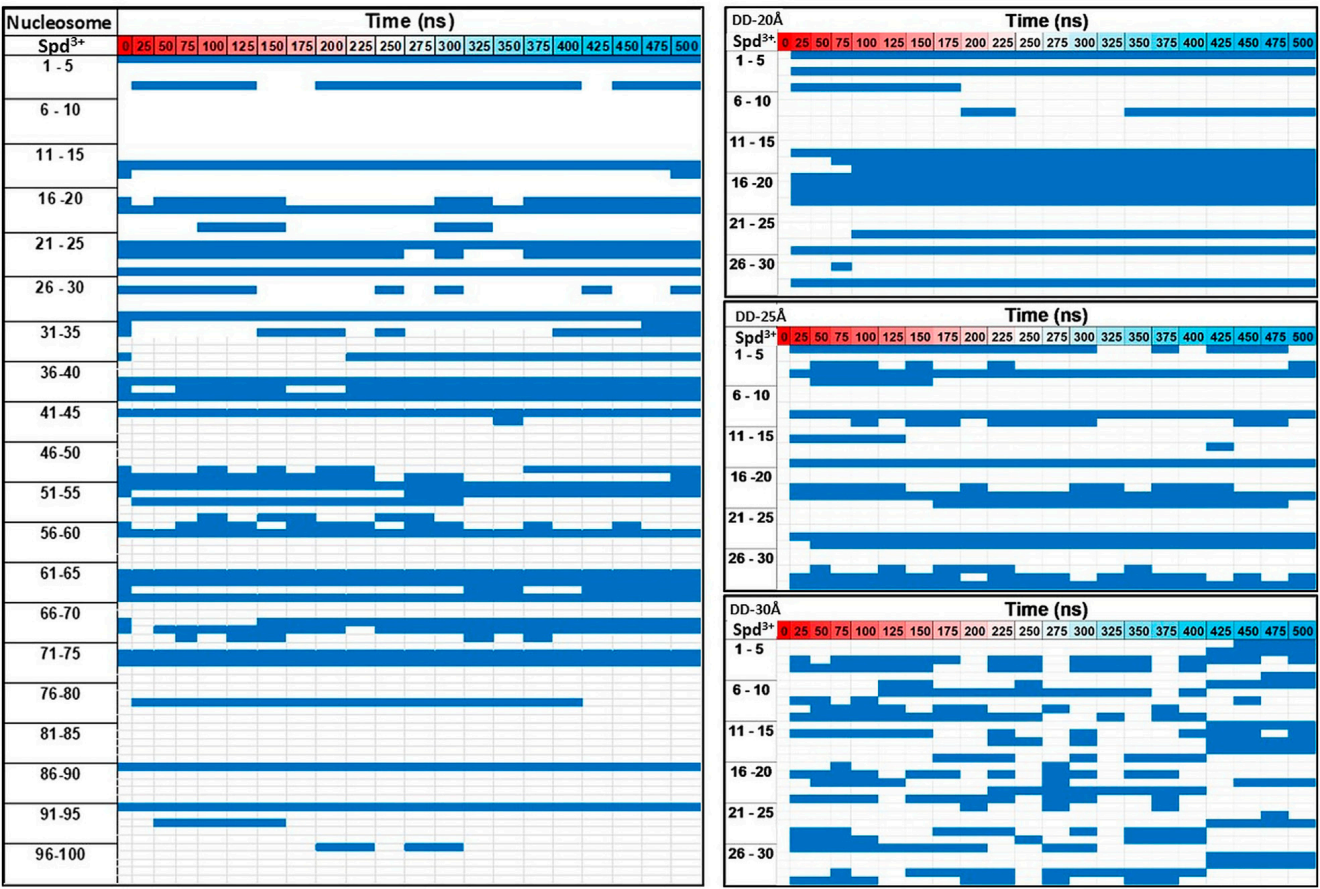
Evolution of the movement of each Spd^3+^ molecule with respect to the caged—uncaged position. The Spd^3+^ are numbered from 1 to 30 for the DD systems and 1 to 100 for the nucleosome system, and the blue corresponds to the caged position.

Next, the dynamics of Spd^3+^ in the caged state was analyzed to better understand how the distance between DNAs effects this property. To this end, we checked the position of each Spd^3+^ (caged or uncaged), sampling configurations at 25 ns intervals over the entire simulation. The results are presented in Figure 8. It can be seen that for the DD-20Å system, once a Spd^3+^ enters the region between the two DNA helices, it effectively remains caged there, i.e. seldom exits from this region. In fact, 11 out of 13 Spd^3+^ stay in the caged position from t = 100 ns (depicted as continuous blue lines in Figure 8), while 15 molecules never reach the caged position. In the DD-25Å system, it can be seen that 7 out of a maximum of 14 molecules stay in the caged position starting from t = 100 ns to the end, while the other caged molecules switch from the caged to the uncaged position several times. Also, there are 13 molecules that never enter the caged region. In the case of the DD-30Å, the Spd^3+^ molecules move between caged and uncaged positions throughout the simulation. Moreover, no molecules remain exclusively in the caged or uncaged state as found in the DD-20Å and DD-25Å simulations. When analyzing the dynamics of Spd^3+^ in the nucleosome system, the similarity to the DD-25Å system becomes even more apparent. The Spd^3+^ dynamics observed in DD-25Å are observed also in the nucleosomal DNA system: part of the Spd^3+^ molecules remain in the caged position for the entire simulation (18 out of 100), others are always in the uncaged position (57 out of 100), while the remainder switch between caged and uncaged states multiple times throughout the entire simulation (25 out of 100). If we consider the ratio between the single-state (i.e. always caged or uncaged) and mixed-state Spd^3+^, a decrease in the ratio of single/mixed-state molecules with increasing distance between the DNA fragments is found: 6.5 for DD-20Å, 2 for DD-25Å and 0 for DD-30Å. Although the nucleosome system has an inter-segment distance of 25-28Å, which places it between the DD-25Å and DD-30Å systems, the ratio of single/mixed-state Spd^3+^ does not follow the same trend, having a value of 3, thus higher than in the DD-25Å system.

On the overall, the Spd^3+^ interactions with DNA are clearly affected by the distance between the DNA molecules, with the increasing confinement between two negatively charged double helices leading to an increase in the concentration of Spd^3+^ in the confined region, and a reduction of the exchange rate of the Spd^3+^ between the caged and uncaged state.

#### 3.3.2 Interaction of Spd^3+^ With the DNA Grooves and Phosphates Groups

To quantify how the overall Spd^3+^ distribution around DNA is affected by the distance between the DNA molecules, the RDFs between Spd^3+^ heavy atoms (i.e., not hydrogen) and DNA were calculated for different regions of the DNA double helices: minor groove (RDF_MIN_), major groove (RDF_MAJ_), and phosphate groups (RDF_PH_). The RDFs averaged over all the nucleotide bases of the DNA molecules are reported and discussed in Supplementary Figures S8, S9, and the corresponding CNs calculated from the integration of the RDFs are reported in Supplementary Table S1. The CNs decrease with increasing DNA-DNA separation from 20 to 25 Å, and then only marginally upon increasing the separation to 30 Å; the corresponding CNs for the nucleosomal DNA system are significantly lower due to the lower Spd^3+^ concentration, which results from our choice to keep the Spd^3+^/phosphate group ratio constant for all the systems studied.

**FIGURE 9:**
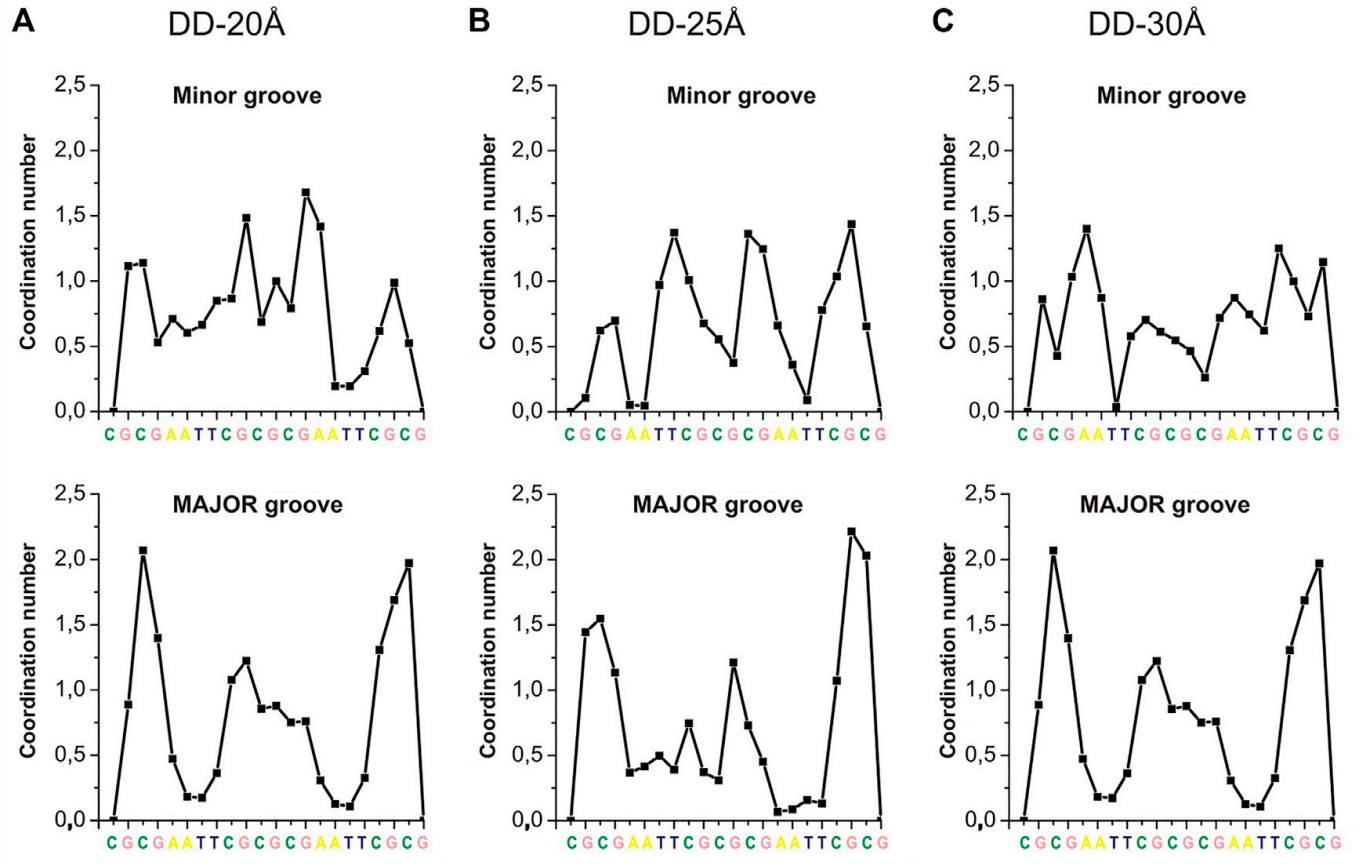
CNs of Spd^3+^ (calculated separately for each DNA base, integrating the corresponding RDFs up to 4.55 Å) in the minor and major grooves of: **(A)** DD-20 Å system; **(B)** DD-25 Å system; **(C)** DD-30Å system.

The RDFs in Supplementary Figures S8, S9 provide only general information about the distribution of Spd^3+^ around DNA. In order to analyze the DNA sequence specificity of Spd^3+^ binding, the CN was calculated separately for each base pair by integrating the corresponding RDF up to 4.55 Å. The dependence of coordination numbers on the nucleotide sequence, averaged over the two DNA helices, are shown in Figure 9. The coordination numbers for each DNA duplex in the system (DNA1 and DNA2) are shown in Supplementary Figure S10. While in previously reported simulations (Perepelytsya et al., 2019) we observed a clear preferential binding of PAs in the minor groove of A-tracts, the sequence specificity of the binding is much less clear in the present simulations (Figures 9A–C). This can be due to several factors: on the one hand, the concentration used in this study is higher, making the sequence specificity less dominant; on the other hand, the presence of multiple charged DNA chains close to each other in a crowded environment strongly affects the electrostatic potential and consequently the interactions. Also, it is possible that the reduced mobility of the Spd^3+^ molecule in the caged state implies longer time to obtain a complete sampling. In the major groove the A-tracts appear to be the less favored binding sites, as also previously observed, due to the steric hindrance of the methyl group of thymine (Perepelytsya et al., 2019). In the grooves of the nucleosome DNA (Supplementary Figure S10) there are large regions which do not interact with Spd^3+^; this is probably due to the fact that the nucleosomal DNA is not allowed to move in this simulation, while dynamical structural rearrangements are necessary for the PAs to enter the grooves, or to find proper coordination modes with the partially negative atoms in these regions.

#### 3.3.3 Modes of Interaction of Spd^3+^ With DNA

**FIGURE 10:**
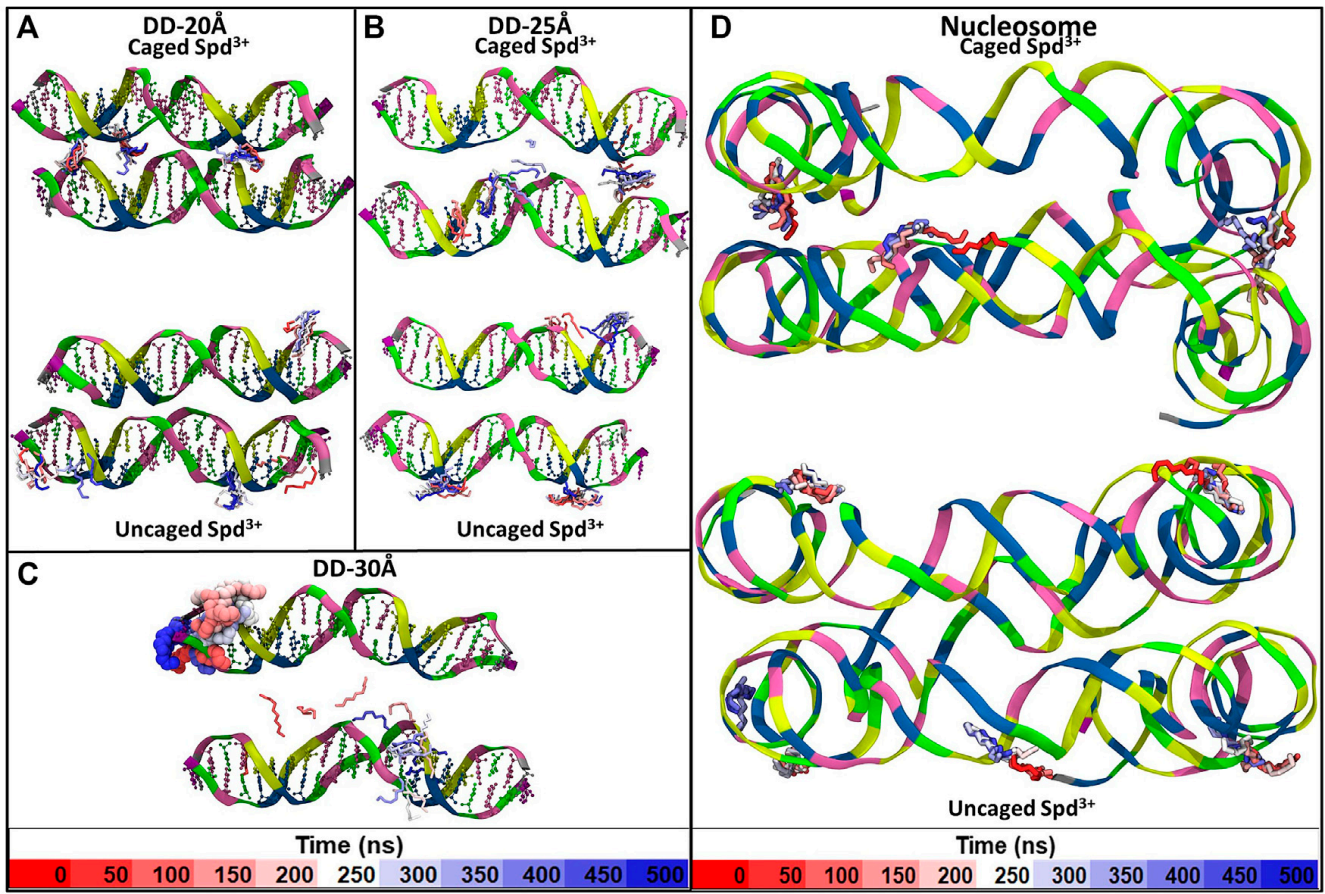
Example of the movement of individual Spd^3+^ over the duration of the simulation of the **(A)** DD-20Å system, **(B)** DD-25Å system, **(C)** DD-30Å system and **(D)** Nucleosome system. For Spd^3+^ we used the licorice representation with H atoms omitted. Each Spd^3+^ changes color from red to white to blue as the simulation time increases, and each change in color corresponds to 50 ns, as shown by the color-coded time scale in the legend. Larger size representations of the Figures are presented in the Supplementary Figures S2–S5.

In addition to the changes in the general mode of interaction of Spd^3+^ with DNA as a function of DNA-DNA distance discussed above, visual analysis of the simulation trajectories reveals changes in the detailed interactions of caged Spd^3+^ molecules. Figure 10 shows selected simulation snapshots of Spd^3+^ molecules in caged and uncaged position. It can be seen that in both the DD-20Å and DD-25Å systems, the uncaged Spd^3+^ behave in one of two ways: a) they remain in close proximity of the same DNA residue throughout nearly the entire simulation, or, b) they move across the surface of the DNA, exploring a larger surface. In the DD-30Å system, all of the Spd^3+^ have a mixed-state distribution, i.e., no Spd^3+^ remains in the caged or uncaged state for the entire simulation. In the DD-20Å system, the caged Spd^3+^ typically remain trapped between the DNA molecules with virtually no movement. In the DD-25Å system, we find that although there are some Spd^3+^ that remain locked in position, other Spd^3+^ molecules can move from one DNA molecule to the other and back, with an intermediate “bridge like” structure (see discussion below). In the DD-30Å system, all Spd^3+^ are moving either along the same DNA molecule or from one DNA molecule to the other forming the same “bridge like” structures. In the nucleosome system, the Spd^3+^ behave in the same way as the DD-25Å system, with some molecules remaining caged in the same position throughout the entire simulation, and other molecules moving along one of the DNA fragments, or from one DNA fragment to the other, as exemplified by the Spd^3+^ on the right in Figure 10D.

**FIGURE 11:**
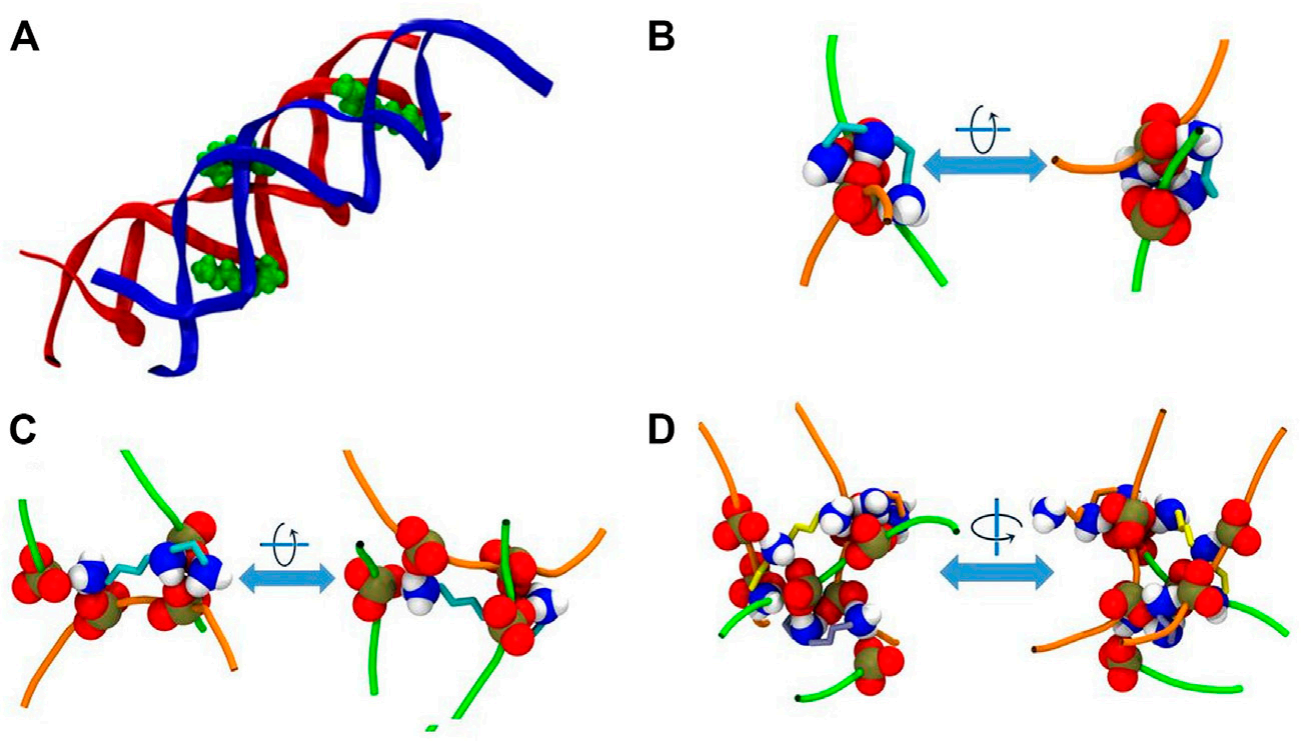
Representative interaction modes of Spd^3+^ with DNA helices. **(A)** Snapshot depicting the parallel-perpendicular (pp) conformation of the Spd^3+^ in the DD-20 Å system. The DNA backbone is represented in red and blue, Spd^3^ green VdW spheres. **(B)** Detail depicting a C-shaped Spd^3+^ that mediates the contact between two phosphate groups, one from each DNA molecules. **(C)** Detail depicting a Spd^3+^ molecule in the pp conformation that mediates the contact between four phosphate groups, two from each DNA molecules. **(D)** Detail depicting a contact point between the two DNA molecules. Three Spd^3+^ mediate this contact point by interacting with 7 PO_3_ groups. In **(B–D)** DNA backbones are depicted as an orange and green tube, one color for each molecule, while the NH_3_, NH_2_ and PO_3_ groups are represented as VdW spheres and the C atoms of Spd^3+^ are represented with licorice in teal, orange, and yellow color. Larger representation of the snapshots can be found in the Supplementary Figures S11–S14.

As stated above, in the system DD-20Å the phosphate groups from different DNA molecules form amino group-mediated contact (O-HNH-O). Visual inspection of the trajectories revealed that several modes of binding of Spd^3+^ to DNA facilitate the close contact between the two DNA helices. Figure 11 depicts various representative interaction modes. In Figure 11A, it can be seen that at the points of contact between the DNAs in system DD-20Å, several Spd^3+^ molecules adopt a “parallel-perpendicular” (pp) orientation, in which the Spd^3+^ are parallel to the backbone of one DNA molecule and perpendicular to the backbone of the second DNA molecule. A detail representation of a Spd^3+^ molecule in the pp orientation simultaneously interacting with 3 DNA strand and 4 phosphate groups, is shown in Figure 11C. It can be seen how this conformation enables the close contacts between the DNA molecules, responsible for the first maximum of the RDF_DD_ and RDF_PH_ discussed above. Figure 11B, depicts another conformation in which Spd^3+^ adopts a C-shape, forming hydrogen bonds with the oxygens of two phosphate groups from different DNA fragments. The amino groups coordinating a given phosphate group often belong to different Spd^3+^ molecules, forming a complex network, as shown in Figure 11D. Here is depicted a contact point between the two DNA molecules and 3 Spd^3+^. It can be seen that the 3 Spd^3+^ molecules interact, each through multiple contacts, with 7 PO_3_ groups. The high local concentration of both positive and negative charged groups, explains why, in the DD-20Å system, the Spd^3+^ caged between the two DNA molecules remains trapped for the entire duration of the simulation as seen in Figure 8.

From Figure 11 we can also see that the Spd^3+^ molecules can adopt different conformations when interacting with DNA; more precisely, rotation around the 7 torsional bonds of this small molecule leads to a wide range of conformations, ranging from a linear conformation to rather compact folded conformation. In order to estimate the probability of different Spd^3+^ conformational states and to determine if the caging alters the distribution among them, we calculated the end-to-end distance (EE) for each Spd^3+^ molecule in the caged and uncaged regions. The EE calculations were performed on configurations obtained by sampling the trajectories every 25 ns, and the EE distributions are represented as histograms in Supplementary Figure S15. In the following, we identify the folded and elongated states as those with EE < 7 Å and EE > 9 Å, respectively. Considering the DD systems, there are only small differences in the EE distributions of the uncaged Spd^3+^ among the three systems: 4–8% are in a folded state, and 46–50% in an elongated state. A different picture emerges when analyzing the caged Spd^3+^ molecules: for the DD-25Å and DD-30Å systems, the EE distributions are similar to those of the uncaged Spd^3+^, with nearly 10% being in a folded state and 44–48% in an elongated state. Conversely, in the case of the DD-20Å system, there is a large increase of the folded state population, which represents 19% of the total, and a decrease to 36% of Spd^3+^ in the elongated state. Therefore, we can conclude that decreasing the distance between DNA molecules induces an increase in the folded states of the caged Spd^3+^ and a decrease in the elongated states, the variations being much larger when the DNA molecules are very close to each other. Considering the nucleosome system, it can be seen that it follows the same trend as the other systems for the uncaged molecules, whereas the caged molecules have a lower probability to be in a folded state (5%) and higher probability to be in an extended state (56%) compared to DD systems. These differences compared to the other systems are most likely due to the constraints applied to the DNA atoms, which prevent the local DNA rearrangement necessary to establish an optimal interaction with the compacted forms of Spd^3+^; this finding suggests that the constraints employed might significantly affect the distribution among different Spd^3+^ conformational states.

## 4 Conclusion

Four model systems, each containing two DNA double helices with different DNA-DNA separation, in the presence of Spd^3+^ and KCl, have been studied using MD computer simulations, with the aim to understand how the separation between DNA double helices influences the interaction with polyamines.

In all the simulated systems, Spd^3+^ molecules bind in all the different regions of the double helix: minor and major grooves and phosphate groups. The presence of a second DNA double helix influences strongly the interactions with Spd^3+^. At small DNA-DNA separation (<25 Å between the helix axes), an increase of Spd^3+^ concentration is observed in the region between the parallel DNA helices, compared to concentration when the helices are further apart (30 Å separation between the axes). The separation between the double helices also affects the modes of interactions of the Spd^3+^ molecule with DNA, indicating that some binding modes accessible when DNA molecules are separated (e.g., those involved in the preferential binding to the minor groove of A-tract in diluted aqueous solution) might not be very accessible in highly compact system (e.g., in the cell nucleus) while other binding modes, involving folded Spd^3+^ configurations could be favored in compact DNA aggregates.

In the most condensed form observed in our simulations, the DNA helices adopt a reciprocal orientation with the DNA-DNA contacts mostly occurring between the minor grooves of the parallel helices. This type of orientation is observed also in the experimental structure of nucleosomal DNA. It is useful to note that to reach this type of arrangement in the simulations, the DNA molecules should be free to rotate around their helix axis, and/or to shift along the same.

The dynamics of the Spd^3+^ molecules are also strongly affected by the DNA-DNA separation: at very small separations (20 Å), the Spd^3+^ located between the DNA molecules remain effectively stuck in their binding sites; increasing the inter-helical separation to 25 Å, the PAs still maintain relatively long residence times in the region between the helices, but they move from one binding site to another. Further increasing the separation between the DNA helices to 30 Å leads to a further increase in Spd^3+^ mobility, thus reducing the residence time in the inter-helical space.

The presented data are of relevance for understanding how the interaction of PAs with DNA in compact systems may differ from those in diluted solution, and to understand the mechanisms of compaction of DNA in biological systems. We wish to add that the effect of DNA compaction on the competition between Spd^3+^ and other counterions found in the solvating shell of nucleic acids in biological systems, e.g., K^+^ (Auffinger and Westhof, 2000; Auffinger and Hashem, 2007; Mocci and Laaksonen, 2012), is an important related topic that will be analyzed in a future study.

## Data Availability

The raw data supporting the conclusion of this article will be made available by the authors, without undue reservation.
