# Supplementary material for: Caging Polycations: Effect of Increasing Confinement on the Modes of Interaction of Spermidine3+ With DNA Double Helices
Source: Front Chem. 2022 Feb 25;10:836994. doi: 10.3389/fchem.2022.836994 (PMC8915389; doi:10.3389/fchem.2022.836994)
Supplement: Supplementary file 1 [file DataSheet1.PDF]

Supplementary Material

Caging polycations: Effect of increasing confinement on the modes of interaction of spermidine^{3+} with DNA double helices

Tudor Vasiliu[1], Francesca Mocci[2*], Aatto Laaksonen[1,2,3,4,5*], Leon De Villiers Engelbrecht[2], Sergiy Perepelytsya[6]

[1] Centre of Advanced Research in Bionanoconjugates and Biopolymers "Petru Poni" Institute of Macromolecular Chemistry, Jasi, Romania

[2] Dipartimento di Scienze Chimiche e Geologiche, Cagliari University, Cagliari, Italy

[3] Energy Engineering, Division of Energy Science, Luleå University of Technology, Luleå, Sweden

[4] Division of Physical Chemistry, Department of Materials and Environmental Chemistry, Arrhenius Laboratory, Stockholm University, Stockholm, Sweden

[5] State Key Laboratory of Materials-Oriented and Chemical Engineering, Nanjing Tech University, Nanjing 211816, China.

[6] Bogolyubov Institute for Theoretical Physics of the NAS of Ukraine, Kyiv, Ukraine

Supplementary Figures and Tables

1.1 Supplementary Figures

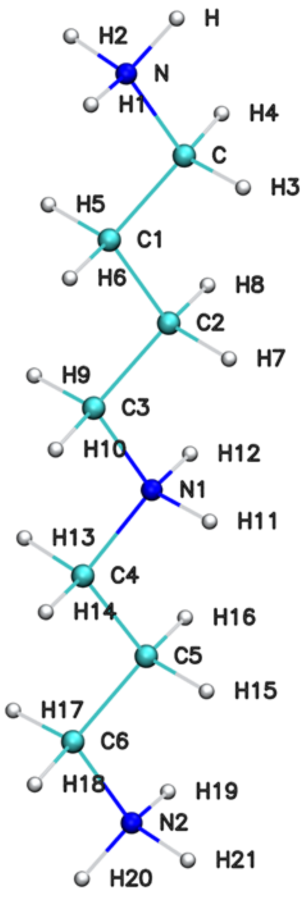

N	-0.3744
H	0.3498
H1	0.3498
H2	0.3498
C	0.0534
H3	0.1186
H4	0.1186
C1	-0.1445
H5	0.0917
H6	0.0917
C2	-0.0179
H7	0.0499
H8	0.0499
C3	-0.0324
H9	0.1118
H10	0.1118
N1	-0.0245
H11	0.266
H12	0.266
C4	-0.0997
H13	0.1148
H14	0.1148
C5	0.0262
H15	0.0466
H16	0.0466
C6	0.0534
H17	0.1186
H18	0.1186
N2	-0.3744
H19	0.3498
H20	0.3498
H21	0.3498

Supplementary Figure 1. Structure and numbering of Spd^{3+} and corresponding atomic charges.

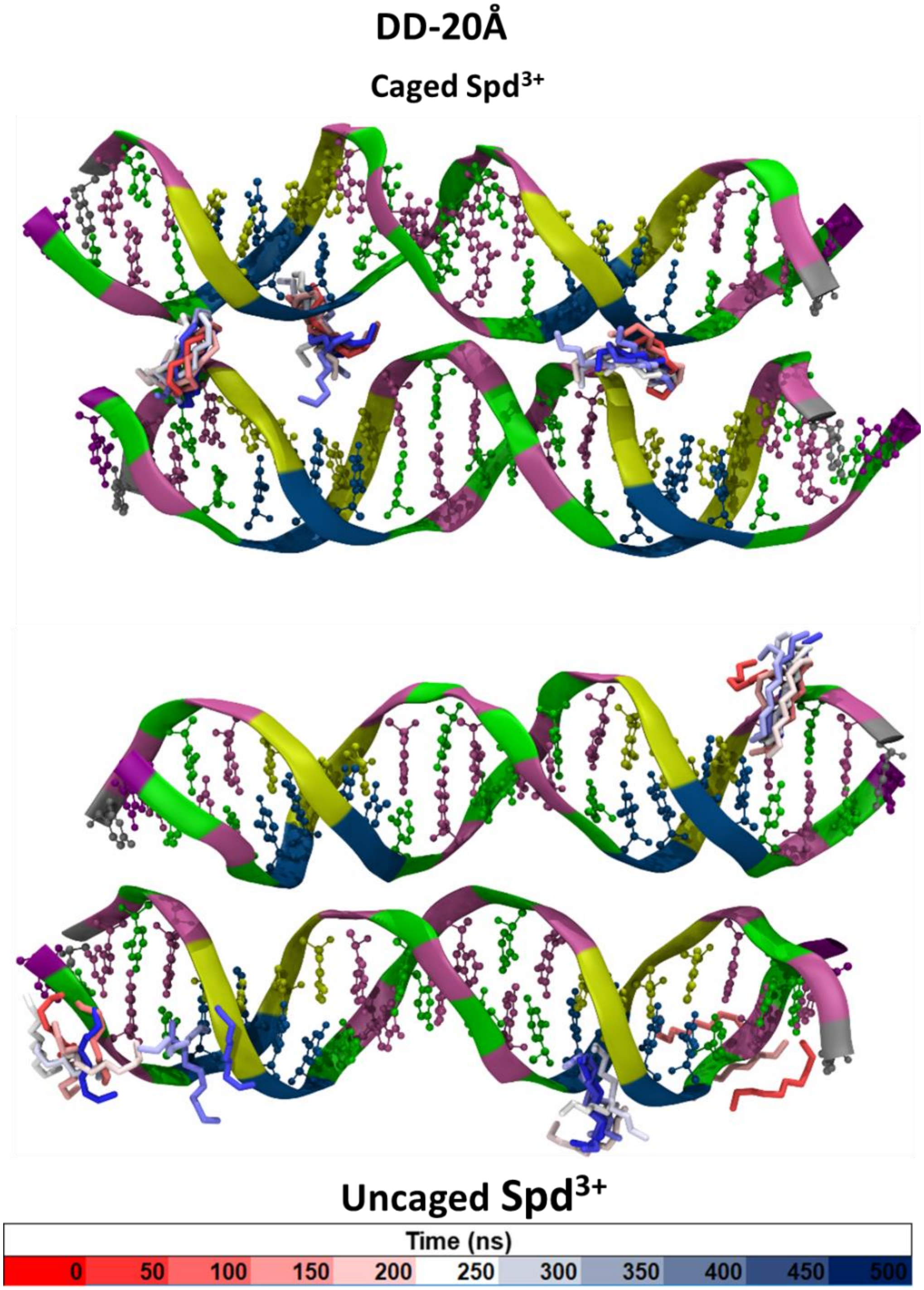

Supplementary Figure 2. Snapshot depicting examples of the trajectory of individual Spd^{3+} molecules over 500 ns for the DD-20Å system. Each Spd^{3+} changes color from red to white to blue as the simulation time increases, and each change in color corresponds to 50 ns. DNA coloring scheme: A, yellow; T, blue; C, green; G, pink.

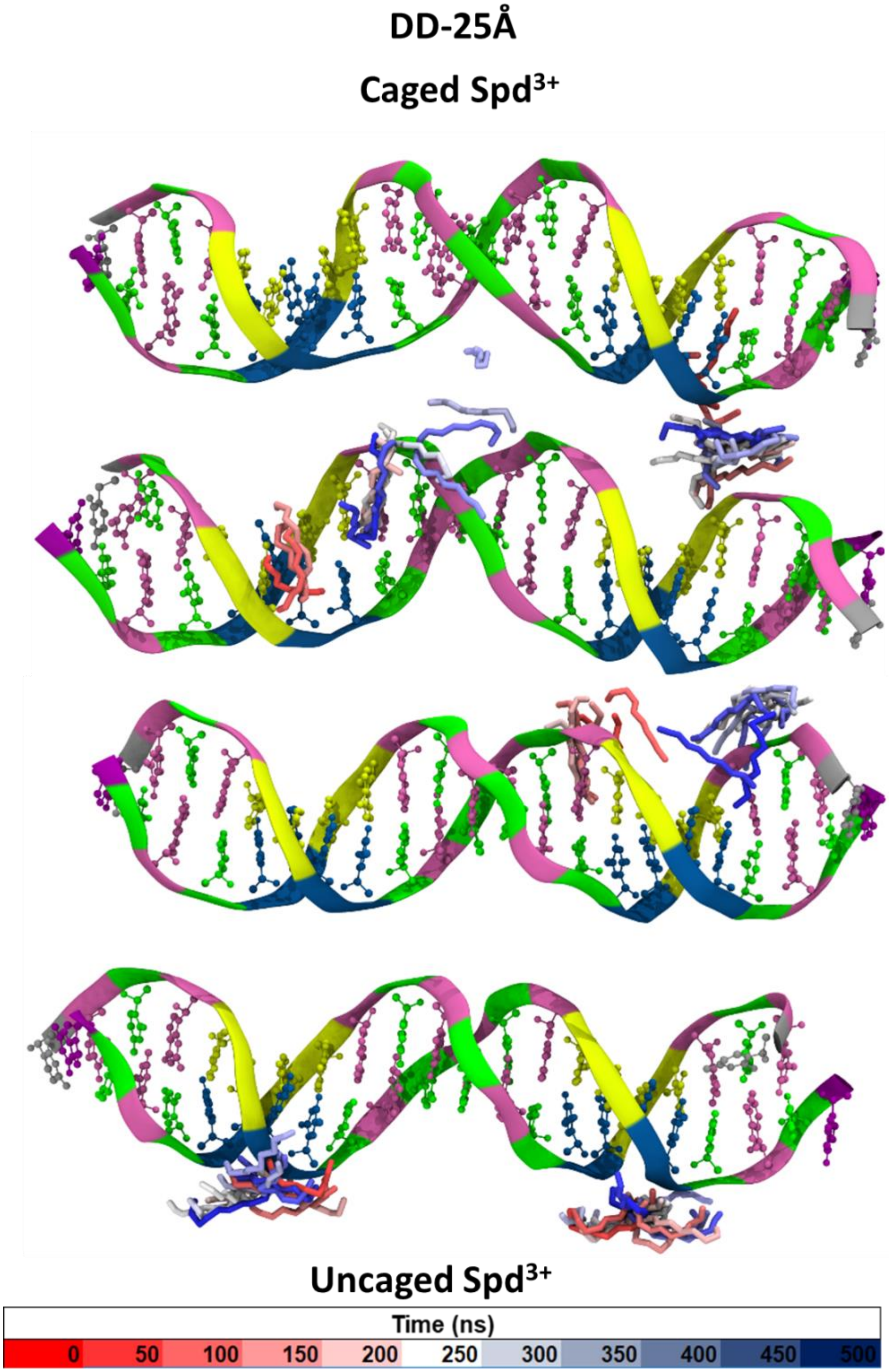

Supplementary Figure 3. Snapshot depicting examples of the trajectory of individual Spd^{3+} molecules over 500 ns for the DD-25Å system. Each spermidine changes color from red to white to blue as the simulation time increases, and each change in color corresponds to 50ns. DNA coloring scheme: A, yellow; T, blue; C, green; G, pink.

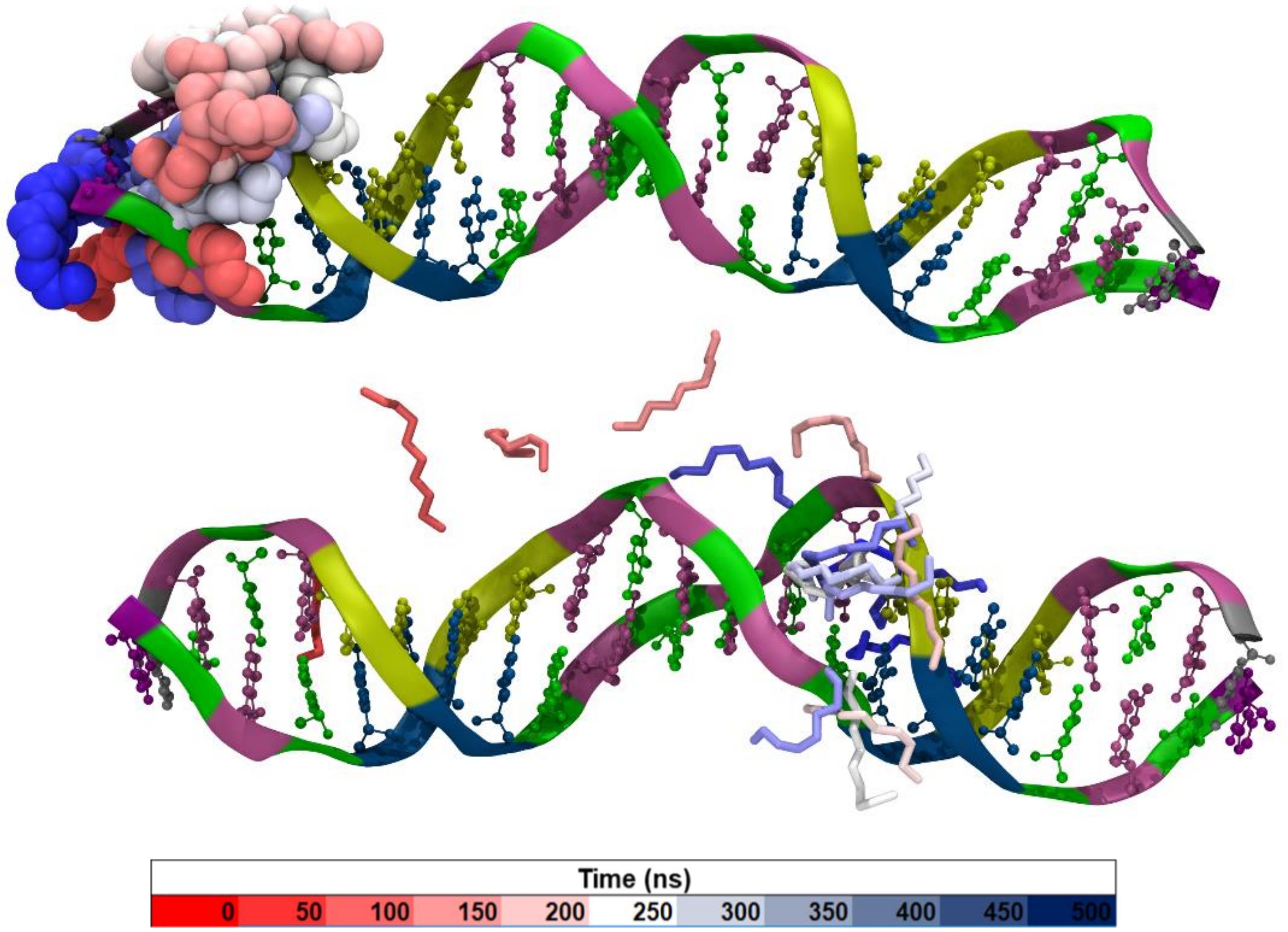

Supplementary Figure 4. Snapshot depicting examples of the trajectories of two individual Spd^{3+} molecules over 500 ns for the DD-30Å system. Each Spd^{3+} changes color from red to white to blue as the simulation time increases, and each change in color corresponds to 50ns, as in Supplementary Figures 2 and 3. The trajectories of the two Spd^{3+} molecules are distinguished by using different representations, van der Waals and liquorice. DNA coloring scheme: A, yellow; T, blue; C, green; G, pink.

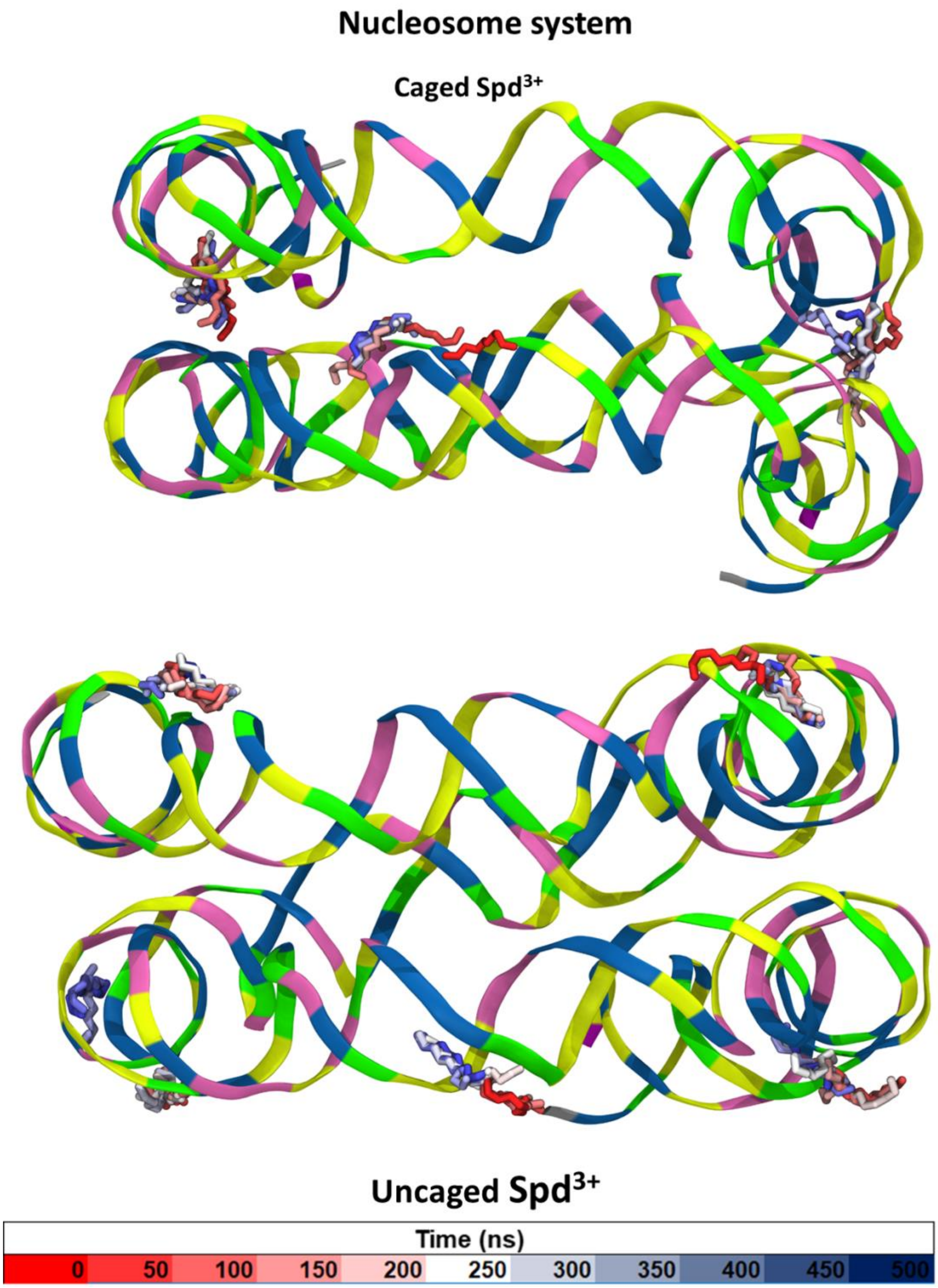

Supplementary Figure 5. Snapshot depicting examples of the trajectory of individual Spd^{3+} molecules over 500 ns for the nucleosome system. Each Spd^{3+} changes color from red to white to blue as the simulation time increases, and each change in color corresponds to 50ns. DNA coloring scheme: A, yellow; T, blue; C, green; G, pink.

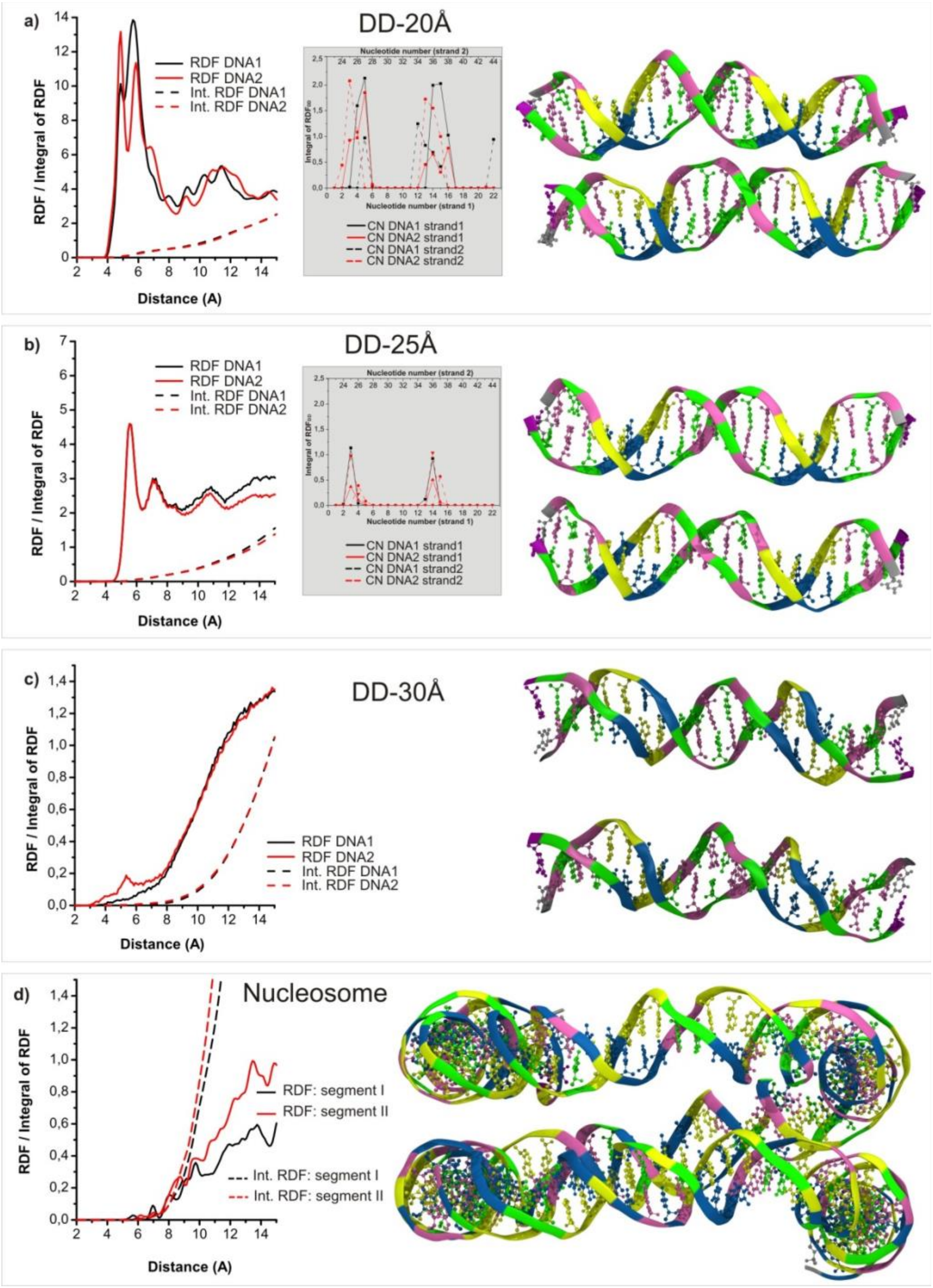

Supplementary Figure 6. Left: RDF_{DD}s (continuous lines) calculated between the P atoms of phosphate groups belonging to different DNA molecules (or parallel segments for nucleosome DNA), and the corresponding integrals (black and red dashed lines). a) System DD-20Å. b) System DD-25Å. c) System DD-30Å. d) Nucleosome DNA system. The insets in (a) and (b) display the values of the integral of the RDFs calculated for each individual phosphate group up to 6.4 Å, revealing the sites were the DNA-DNA contacts are most probable. **Right:** A snapshots from the MD simulation of each system is represented at the right side of the corresponding RDF. Coloring scheme: A, yellow; T, blue; C, green; G, pink. Spd^{3+}, water and ions have been omitted for clarity.

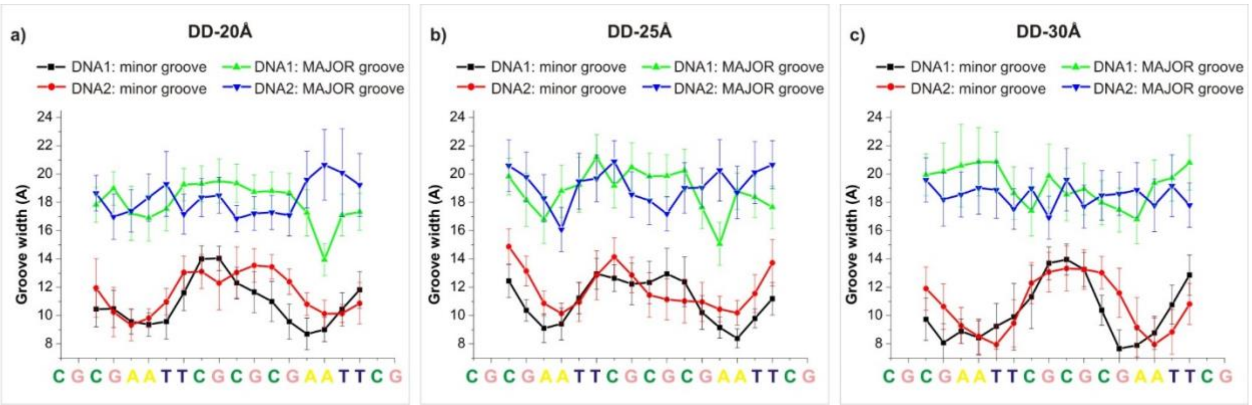

Supplementary Figure 7. Width of the minor and major grooves for each base pair, in a) DD-20Å, b) DD-25Å, c) DD-30Å.

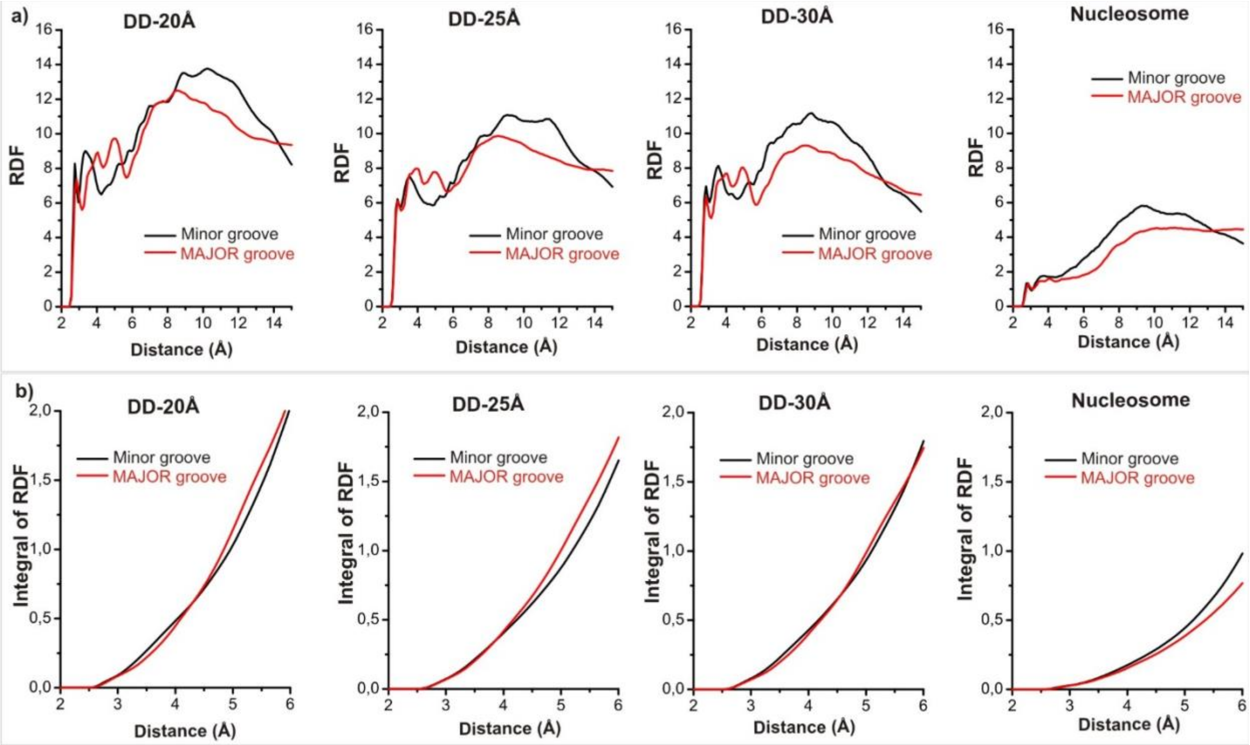

Supplementary Figure 8. a) RDF_{MIN} and RDF_{MAJ} and b) corresponding integrals of all heavy atoms Spd^{3+} with respect to selected atoms of the minor and major grooves (see Figure 1b of the main manuscript) for DD-20Å, DD-25Å, DD-30Å and nucleosome systems. The RDFs and their integrals were averaged over DNA1 and DNA2. The RDF_{MIN} and RDF_{MAJ} are characterized by two local maxima at close distances (below ~4.5 Å) and a broad, convoluted band between ~6 to 14 Å. The former maxima are due to Spd^{3+} in close contact with DNA, and their integration provides average Spd^{3+} CNs around the minor and major grooves, respectively. The CNs, reported in Table S1, decrease with increasing DNA-DNA separation from 20 to 25 Å, and then only marginally upon increasing the separation to 30 Å; the corresponding CNs for the nucleosomal DNA system are significantly lower due to the lower Spd^{3+} concentration, which results from our choice to keep the Spd^{3+}/phosphate group ratio constant for all the systems studied.

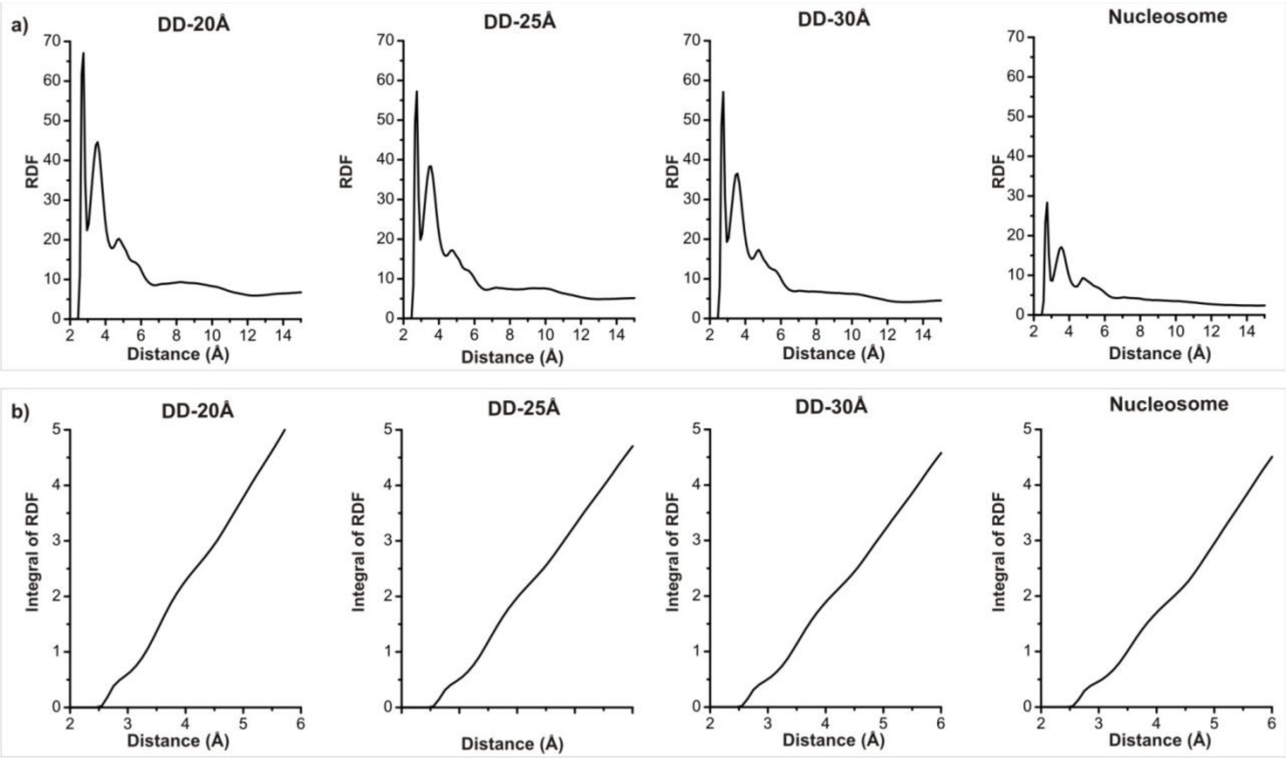

Supplementary Figure 9. a) RDF_{PH} and b) corresponding integrals of Spd^{3+} heavy atoms and the oxygen atoms of the phosphate groups in the case of DD-20Å, DD-25Å, DD-30Å and nucleosome systems. The RDFs and their integrals were averaged over DNA1 and DNA2. For all the studied systems, the RDF_{PH}s are characterized by two maxima and a shoulder separated by about 1 Å distance. The first maximum is due to Spd^{3+} atoms in direct contact with DNA, while the second maximum originates primarily from the Spd^{3+} atoms bound to those in direct contact with phosphate groups. As observed for the interactions in the grooves, the intensity of the first maximum, and the value of the corresponding CNs reported in the Supplementary Table 1, is higher in DD-20Å compared to DD-25Å, and no relevant decrease is observed in DD-30Å compared to DD-25Å. On the overall, this indicates increasing interactions with Spd^{3+} with increasing compaction of the system, due to the increased number of close interactions with the caged PAs.

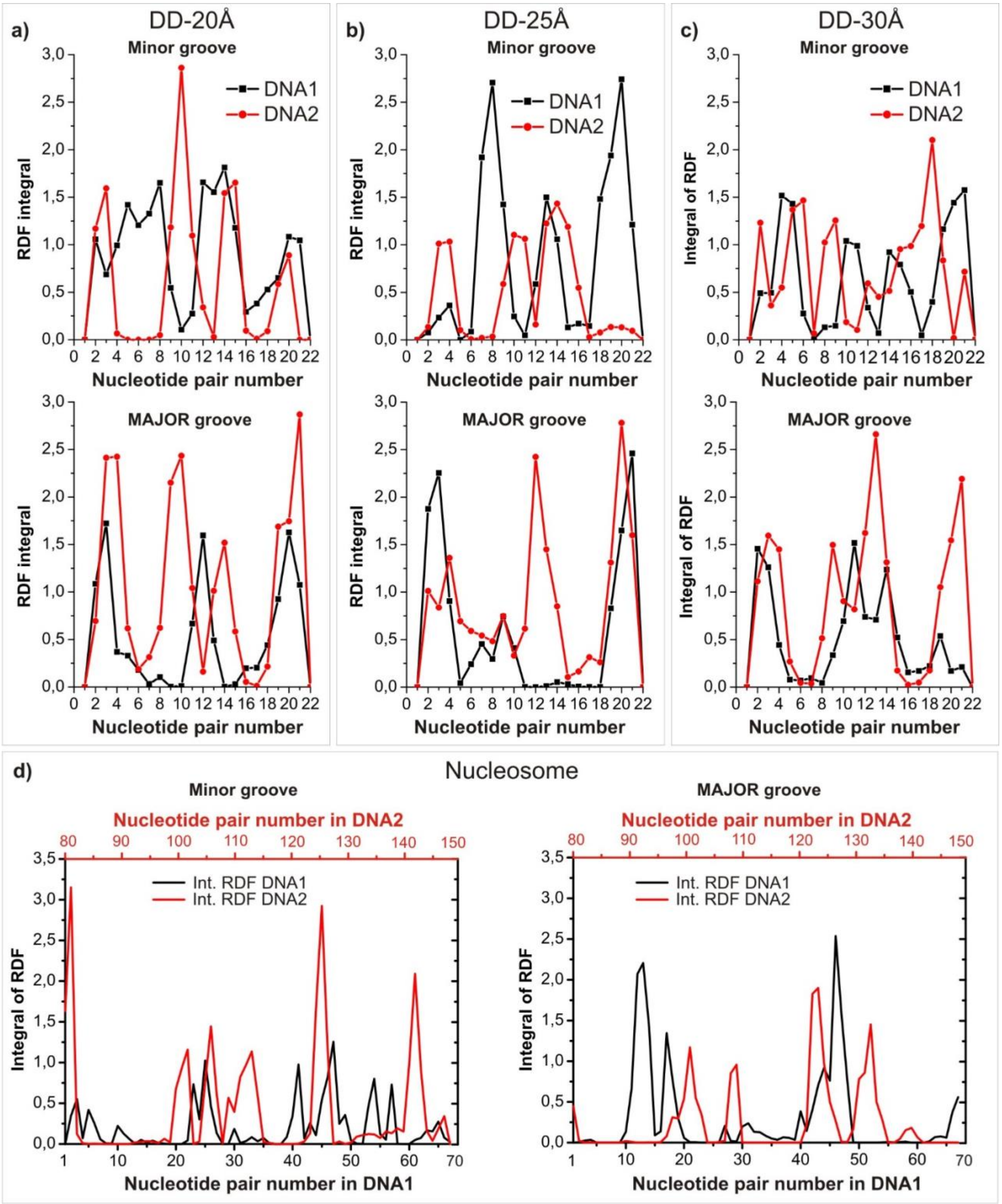

Supplementary Figure 10. CNs of Spd^{3+} (calculated up to 4.55 Å) in the minor and major grooves of a) DD-20Å system; b) DD-25Å system; c) DD-30Å system; d) nucleosome system (the part I of DNA is DNA1 and part II of DNA is DNA2).

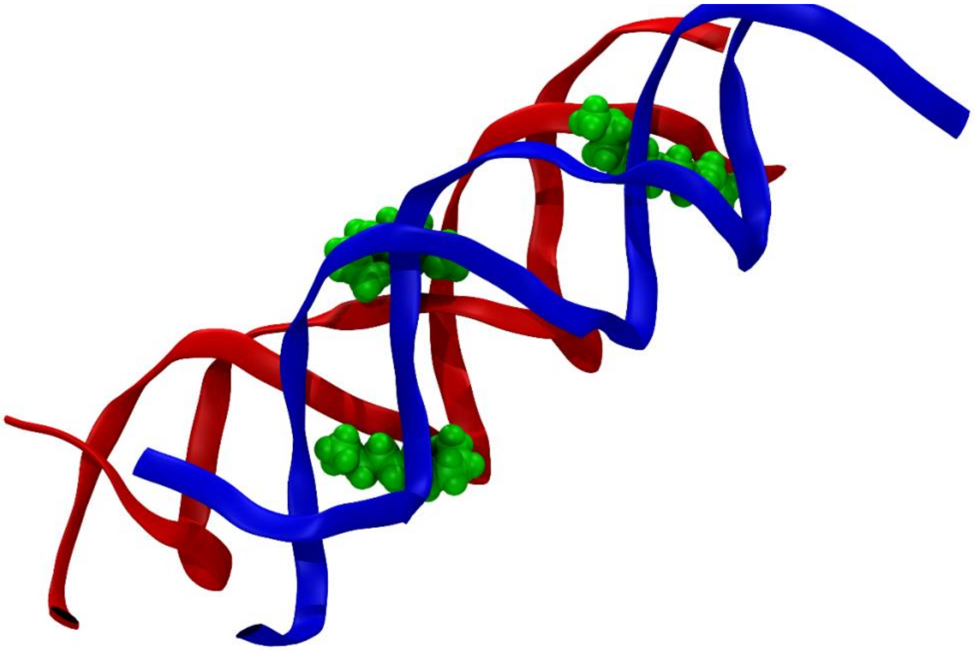

Supplementary Figure 11. Snapshot depicting the parallel-perpendicular (pp) conformation of the Spd^{3+} in the DD-20Å system. The DNA1 and DNA2 backbones are represented in red and blue respectively, and the Spd^{3+} is represented in green VdW spheres. All other atoms in the simulation are omitted for clarity.

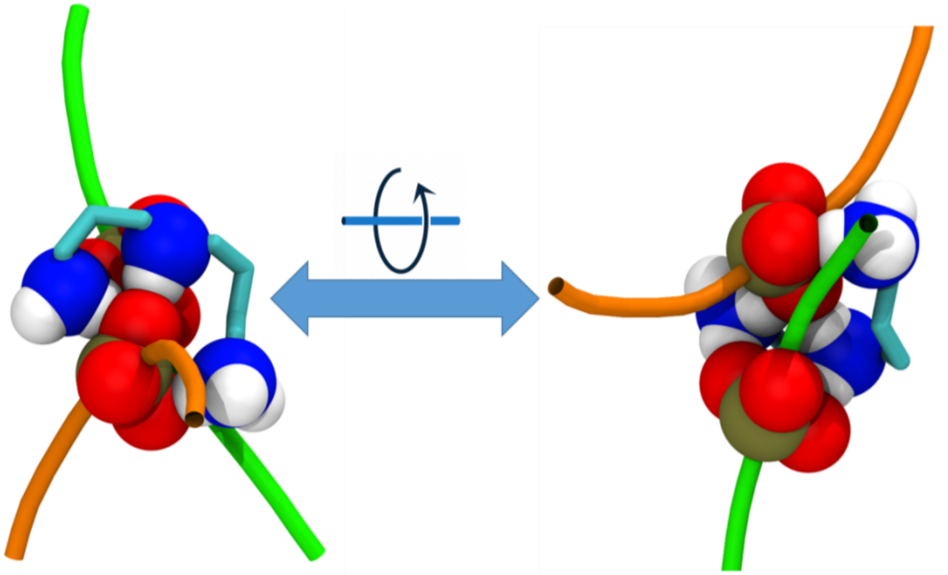

Supplementary Figure 12. Detail depicting a C-shaped Spd^{3+} that mediates the contact between two phosphate groups, one from each DNA molecules. The DNA1 and DNA2 backbones are depicted as an orange and green tubes, respectively. The NH_3, NH_2 and PO_3 groups are represented as VdW spheres and the C atoms of Spd^{3+} are represented with licorice in teal color.

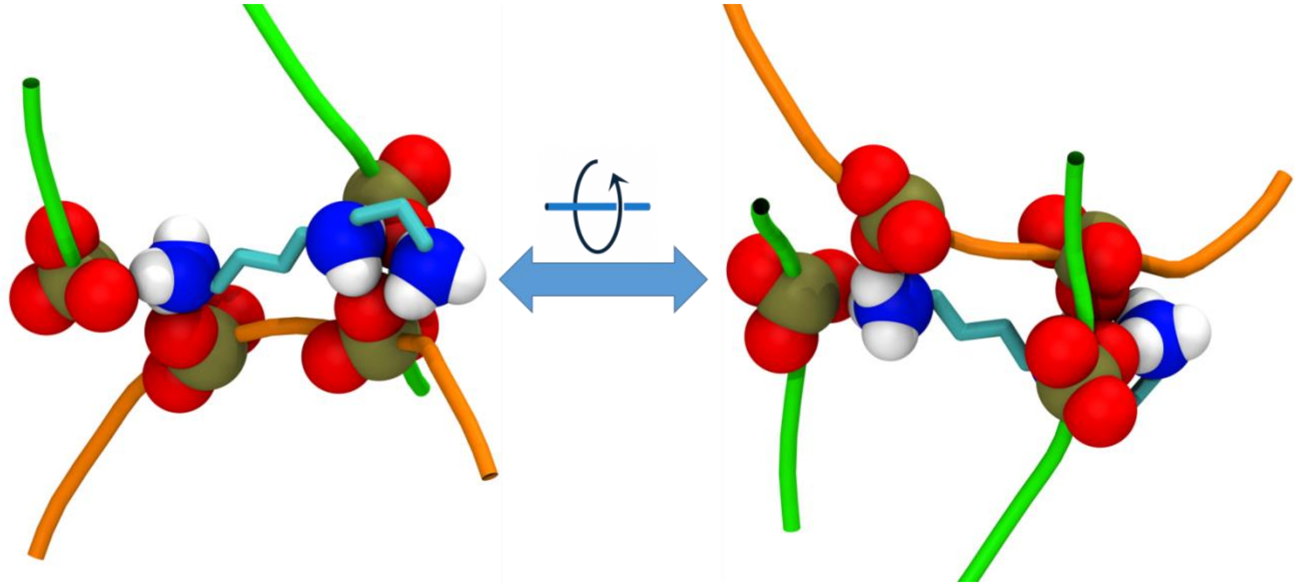

Supplementary Figure 13. Detail depicting a Spd^{3+} molecule in the pp conformation that mediates the contact between four phosphate groups, two from each DNA molecules. The DNA1 and DNA2 backbones are depicted as an orange and green tubes, respectively. The NH_3, NH_2 and PO_3 groups are represented as VdW spheres and the C atoms of Spd^{3+} are represented with licorice in teal color.

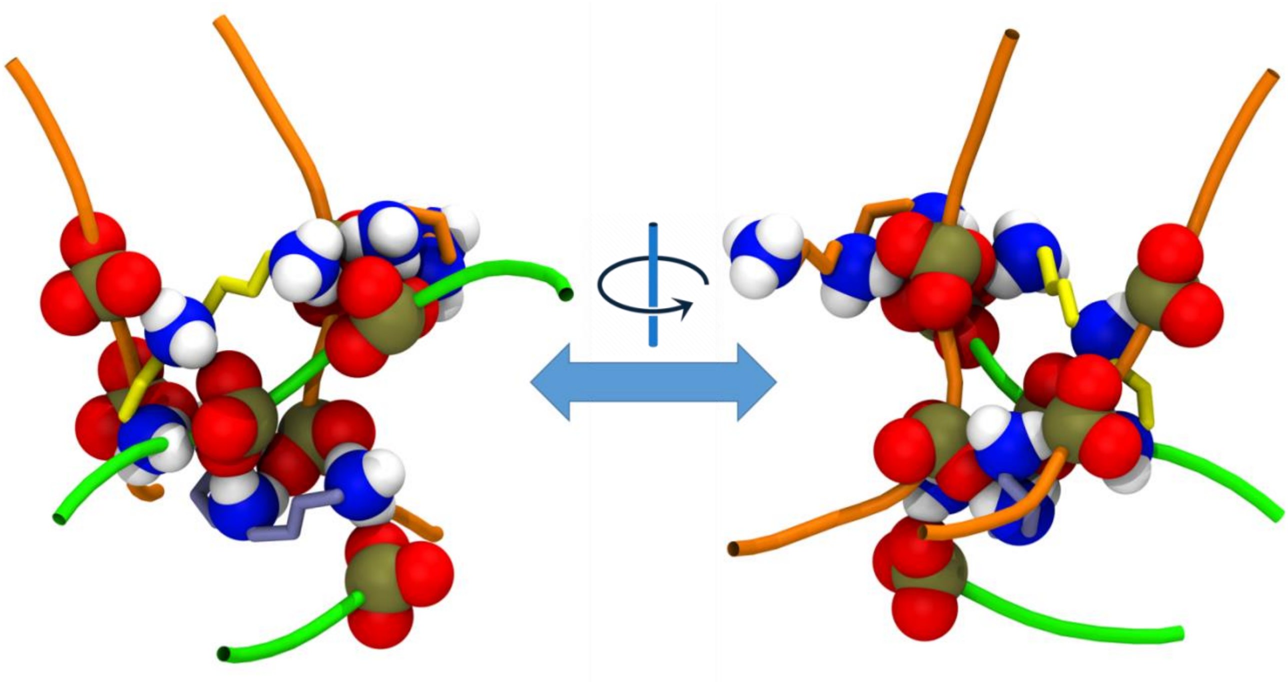

Supplementary Figure 14. Detail depicting a contact point between the two DNA molecules. Three Spd^{3+} mediate this contact point by interacting with 7 PO_3 groups. The DNA1 and DNA2 backbones are depicted as an orange and green tubes, respectively. The NH_3, NH_2 and PO_3 groups are represented as VdW spheres and the C atoms of Spd^{3+} are represented with licorice in teal, orange, and yellow color.

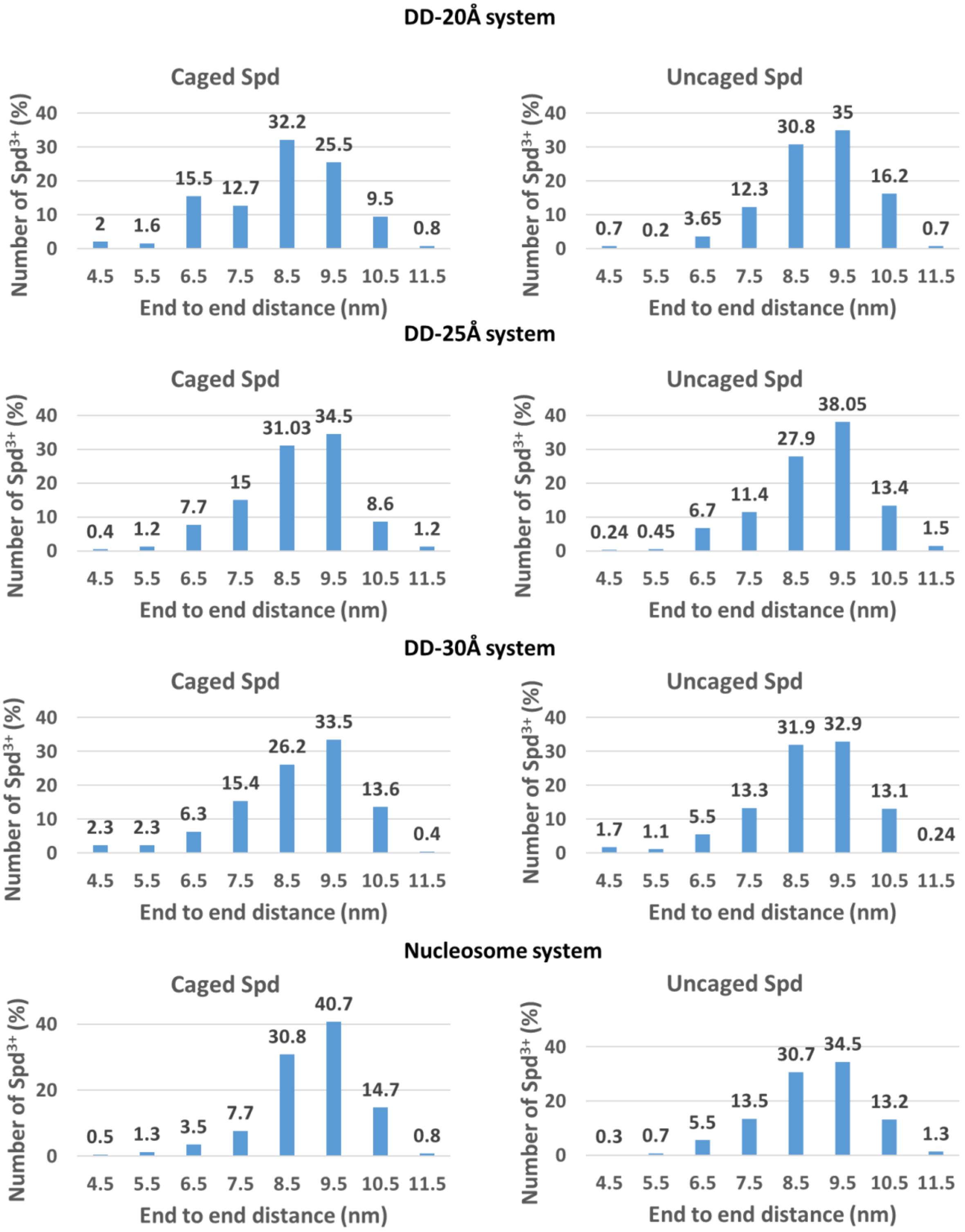

Supplementary Figure 15 Distribution of end-to-end distance of the caged (left) and uncaged (right) Spd^{3+} molecules.

Supplementary Table

Supplementary Table 1. The values of integrals of RDFs (coordination numbers, CN) for heavy atoms of Spd^{3+} at two different distances (3.05, 4.55 Å) from the reference atoms. The values are shown for RDF_{MAJ}, RDF_{MIN}, RDF_{PH}.

System	Integration distance (Å)	RDF_{MIN}	RDF_{MAJ}	RDF_{PH}
DD-20Å	3.05	0.28898	0.23748	1.47543
	4.55	0.74354	0.76975	3.01055
DD-25Å	3.05	0.08246	0.07865	0.54844
	4.55	0.64137	0.69952	2.62201
DD-30Å	3.05	0.08944	0.08032	0.53965
	4.55	0.67655	0.67575	2.5033
Nucleosome	3.05	0.03186	0.03004	0.49429
	4.55	0.30177	0.26596	2.26717
